# Protocol of a randomised controlled trial of real-time continuous glucose monitoring in neonatal intensive care ‘REACT’

**DOI:** 10.1136/bmjopen-2017-020816

**Published:** 2018-06-04

**Authors:** Kathryn Beardsall, Lynn Thomson, Catherine Guy, Mirjam M van Weissenbruch, Isabel Iglesias, Priya Muthukumar, Sateesh Kumar Somisetty, Simon Bond, Stavros Petrou, David Dunger, REACT Investigators, David Dunger

**Affiliations:** 1 Department of Paediatrics, University of Cambridge, Cambridge, UK; 2 Department of Paediatrics and Neonatology, Cambridge University Hospitals NHS Foundation Trust, Cambridge, UK; 3 Department of Paediatrics, VU University Medical Center, Amsterdam, The Netherlands; 4 Department of Paediatrics, Sant Joan de Déu, Barcelona, Catalunya, Spain; 5 Department of Paediatrics, Norfolk and Norwich University Hospitals NHS Foundation Trust, Norwich, UK; 6 Department of Paediatrics, Luton and Dunstable University Hospital, Luton, UK; 7 Cambridge Clinical Trials Unit, Cambridge University Hospitals NHS Foundation Trust, Cambridge, UK; 8 Division of Health Sciences, Warwick Medical School, University of Warwick, Coventry, UK

**Keywords:** randomised controlled trial, glucose, continuous monitoring, hyperglycaemia, hypoglycaemia

## Abstract

**Introduction:**

Hyperglycaemia is common in the very preterm infant and has been associated with adverse outcomes. Preventing hyperglycaemia without increasing the risk of hypoglycaemia has proved challenging. The development of real-time continuous glucose monitors (CGM) to inform treatment decisions provides an opportunity to reduce this risk. This study aims to assess the feasibility of CGM combined with a specifically designed paper guideline to target glucose control in the preterm infant.

**Methods and analyses:**

The Real Time Continuous Glucose Monitoring in Neonatal Intensive Care (REACT) trial is an international multicentre randomised controlled trial. 200 preterm infants ≤1200 g and ≤24 hours of age will be randomly allocated to either real-time CGM or standard care (with blinded CGM data collection). The primary outcome is time in target 2.6–10 mmol/L during the study intervention assessed using CGM. Secondary outcomes include efficacy relating to glucose control, utility including staff acceptability, safety outcomes relating to incidence and prevalence of hypoglycaemia and health economic analyses.

**Ethics and dissemination:**

The REACT trial has been approved by the National Health Service Health Research Authority National Research Ethics Service Committee East of England (Cambridge Central); Medical Ethics Review Committee, VU University Medical Centre, Amsterdam, The Netherlands and the Research Ethics Committee, Sant Joan de Déu Research Foundation, Barcelona, Spain. Recruitment began in July 2016 and will continue until mid-2018. The trial has been adopted by the National Institute of Health Research Clinical Research Network portfolio (ID: 18826) and is registered with anInternational Standard Randomised Control Number (ISRCTN registry ID: 12793535). Dissemination plans include presentations at scientific conferences, scientific publications and efforts at stakeholder engagement.

**Trial registration number:**

ISRCTN12793535; Pre-results.

**Strengths and limitations of this study**

- The comparison of real-time continuous glucose monitoring (CGM) data with blinded CGM data in the control study arm will provide detailed comparable data on efficacy and safety between study arms.
- As an international multicentre trial, the results will be generalisable across a range of neonatal intensive care settings.
- Input by staff and parents within the trial itself as well as part of the trial management will provide information on utility and facilitate translation of the outcomes into clinical practice.
- The study requires recruitment within 24 hours of preterm birth which requires a significant commitment from the clinical and research teams if it is to be successful.
- The study is powered to detect a difference in the primary outcome ‘time in target’ (2.6–10 mmol/L), but will not have the power to detect the impact on clinical outcomes.

## Introduction

In utero, glucose levels are normally maintained between 4 and 6 mmol/L,^1^ but infants born preterm are at risk of both hyperglycaemia and hypoglycaemia.^2^ Hyperglycaemia and hypoglycaemia have both been associated with increased mortality and morbidity of preterm babies.^3 4^ Hyperglycaemia can lead to acute problems of a persistent osmotic diuresis and metabolic acidosis which can be difficult to control and has been associated with increased risk of intraventricular haemorrhage and patent ductus arteriosus.^5^ Hyperglycaemia has also been associated with increased long-term morbidity, including increased risk of retinopathy of prematurity.^4 6–8^ Hypoglycaemia is associated with characteristic occipital temporal lesions.^8^ Attempts to reduce risks associated with hyperglycaemia in adult and paediatric and neonatal intensive care (NICU) have resulted in increased risk of hypoglycaemia.^9–11^ This is of particular concern for the very preterm infant in whom there is very varied insulin sensitivity, which increases the risk of hypoglycaemia. In addition the developing brain appears to be particularly vulnerable to both hyperglycaemic^12^ and hypoglycaemic insults. Early postnatal glucose control may be an important modifiable risk factor for clinical outcomes. A recent Cochrane review has highlighted the need for further studies into the impact of interventions to improve glucose control in these infants.^13^

Managing glucose control is dependent in part on methods of measuring and monitoring glucose levels. Within NICU glucose measurements are currently limited to intermittent blood sampling,^14^ with long periods when glucose levels are unknown. In contrast, other physiological parameters such as oxygen saturation, blood pressure and heart rate are all monitored continuously to prevent wide fluctuations. It is increasingly thought that fluctuations in glucose levels may also have a significant impact on long-term outcomes.^15^ The reason for the intermittent measurement is that current methodologies rely on blood sampling either from a central arterial line or by heel prick. Clinical care for preterm infants aims to reduce the frequency of handling^16^ and volume of blood sampled as this has been shown to improve outcomes.

Developments in the measurement of glucose levels in patients with diabetes mellitus include continuous glucose monitoring (CGM) of interstitial glucose levels.^17^ Real-time data on interstitial glucose levels now provides information on glucose trends with the potential for earlier intervention and prevention of both hyperglycaemia and hypoglycaemia. Benefits within adult intensive care remain controversial,^18^ however, the benefits in the setting of NICU may be more marked as blood glucose (BG) measurements are taken much less frequently in these small babies. There have also been key developments in the technology including extended life of sensors (previously 72 hours) and improved accuracy.^19^ The latter is particularly relevant due to the threshold levels of hypoglycaemia and hyperglycaemia (<2.6 and >10 mmol/L) which are more extreme than in adults and are at the limits of accuracy of many methods of glucose measurement.

Blinded CGM has been used in preterm babies within clinical trials,^2 20 21^ and studies of real-time devices have shown a benefit in providing early warning of and prevention of hypoglycaemia.^22^ Studies have not so far attempted to use CGM to guide clinical management to support the targeting of glucose control in preterm infants. A single-centre feasibility study of the real-time monitors demonstrated that sensor glucose (SG) values are comparable with BG values (Real Time Continuous Glucose Monitoring in Neonatal Intensive Care (REACT) feasibility study REC Ref: 14/EE/0127). Data from the feasibility study have helped to design this randomised controlled trial. This multicentre randomised controlled trial will determine whether real-time CGM, with support from a paper algorithm, can help improve the management of glucose control in terms of efficacy, safety and clinical acceptability. This will not only enhance the short-term management of glucose control in infants requiring intensive care but by reducing the risks associated with both hyperglycaemia and hypoglycaemia may impact on long-term clinical outcomes.

## Trial objectives

The REACT trial will evaluate efficacy, safety, utility and cost-effectiveness of real-time CGM in preterm infants in NICU. Our primary hypothesis is that the use of real-time CGM will improve the time a baby’s glucose levels (measured using CGM) remain within the target 2.6–10 mmol/L (widely accepted clinical target for glucose control), compared with standard clinical practice (with blinded CGM data collection). Secondary objectives include evaluation of clinical acceptability in the preterm infant (using staff and parent questionnaires) and safety in relation to the device itself and risk of hypoglycaemia.

## Methods

### Study design

This is a multicentre interventional, randomised controlled trial of CGM compared with standard clinical management (control). We will recruit potential participants within 24 hours of birth and continually monitor their glucose levels for 6 days. Data will be collected until 36 weeks corrected gestational age. No other aspects of concomitant care are prohibited during the trial.

### Eligibility criteria

#### Inclusion criteria

Infants who have a birth weight ≤1200 g, are ≤24 hours of age, ≤33+6 weeks gestation and in whom written informed parental consent has been received.

#### Exclusion criteria

Any lethal congenital abnormality known at trial entry, any congenital metabolic disorder known at trial entry and any neonates who, in the opinion of the treating clinician at trial entry, have no realistic prospect of survival.

### Intervention

Babies will be randomised in a 1:1 ratio, into control and intervention arms of the study using a web randomisation system, Trans European Network ALEA software (TENALEA). This is an open study in which the clinical staff, research team and parents will be aware of the study arm and intervention. All babies will have a subcutaneous sensor inserted, Enlite (Medtronic) that will be linked to a Medtronic MiniMed 640G system and will be calibrated with point of care BG levels. For consistency across sites calibration will be standardised by providing all units with Nova StatStrip meters.

#### Standard care with blinded CGM data collection (control)

These infants will have their glucose control monitored and managed according to standard clinical practice using intermittently sampled BG levels. Nutritional delivery (including glucose) and insulin delivery will be prescribed according to the standard clinical guidelines within each unit. The CGM device will collect glucose data continuously, but the clinical team will be blinded to the data as the monitor will be kept covered and fastened with a tamper proof seal.

#### Real-time CGM device with paper algorithm (intervention)

The CGM data will be open to view by the clinical team during the first week of the baby’s life and the staff will be advised to read and record the SG data hourly as part of standard clinical monitoring. This will support staff to use the additional data available from real-time monitoring to guide timing of BG measurement and changes in clinical management. Interventions to target glucose control will then be guided by the specifically designed paper algorithm (Supplementary file). This algorithm was developed during the REACT feasibility study (REC Ref: 14/EE/0127).

10.1136/bmjopen-2017-020816.supp1 Supplementary file 1

### Medical devices: MiniMed 640G system

The MiniMed 640G system is indicated for glucose monitoring and for continuous delivery of insulin, for the management of diabetes mellitus in persons requiring insulin. Monitoring equipment only will be used for this study. The system being used comprises linking the Enlite sensor (Medtronic, Northridge, California, USA) using the Guardian 2 Link transmitter to the MiniMed 640G which then displays the glucose data in real time.

#### Sensor

**Figure 1:**
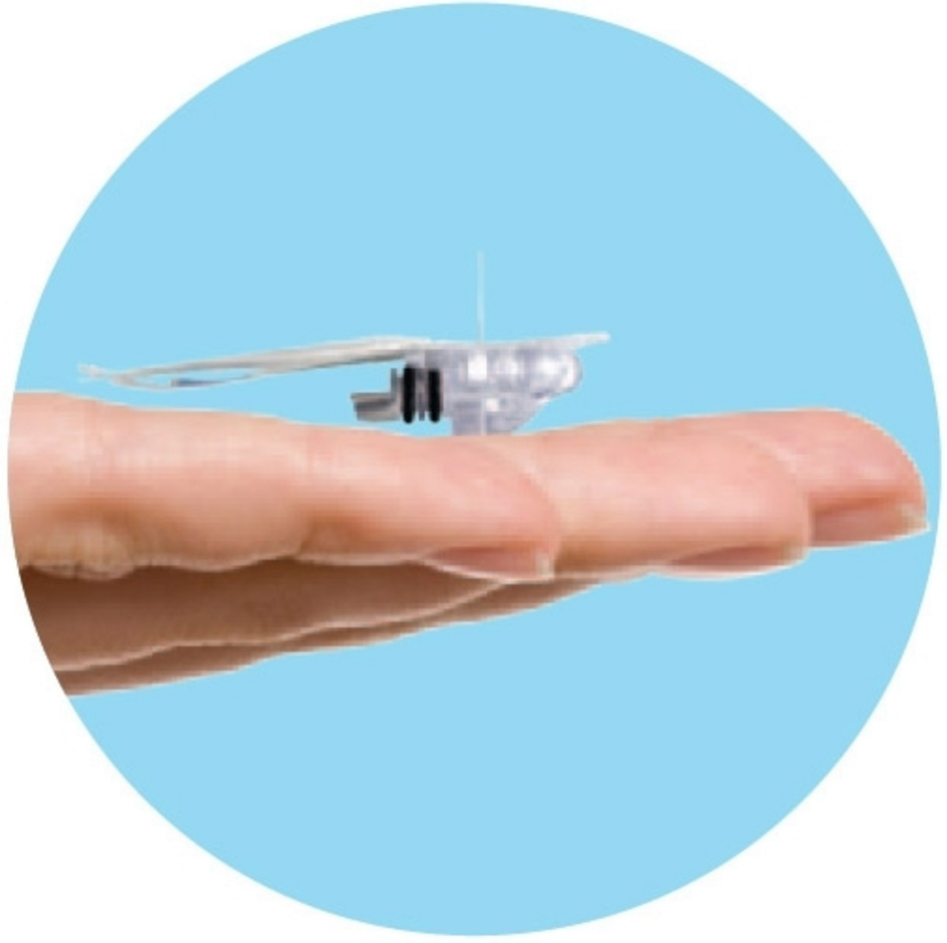
The Enlite glucose sensor (Medtronic, Watford UK).

The Enlite sensor (Medtronic) is a CGM sensor which received CE mark in 2013 (CE certificate No. 21024). The sensor (figure 1) comprises a disposable subcutaneous oxidase-based platinum electrode that catalyses interstitial glucose generating an electrical current every 10 s which is transmitted to a monitor for display and/or recording. The data will be recorded and/or displayed as an averaged value every 5 min, giving a total of 288 readings per day. Glucose values outside the range 2.2–24.0 mmol/L (40–430 mg/dL) are reported as <2.2 mmol/L (40 mg/dL) or >24 mmol/L (430 mg/dL), respectively.

The sensor will be inserted subcutaneously (into the thigh) by hand, not using the standard insertion device, thus ensuring the sensor is inserted into the subcutaneous tissue. The sensors are soft and flexible, approximately 8.75 mm in length and are mounted inside a hollow needle to allow for subcutaneous insertion. Once the sensor is inserted the introducer needle will be withdrawn, and the sensor attached to a small Guardian 2 Link transmitter (CE Mark 2013; Certificate No. 8858) for data transfer to the MiniMed 640G system for data viewing. The sensor will then be secured with a clear occlusive dressing (again trimmed to ensure minimal contact with the infant’s skin), so that the insertion site can be inspected daily. A blood sample will be required in every 12 hours to ensure calibration of the sensor. Sensors will be removed after 7 days.

#### Guardian 2 Link transmitter

**Figure 2:**
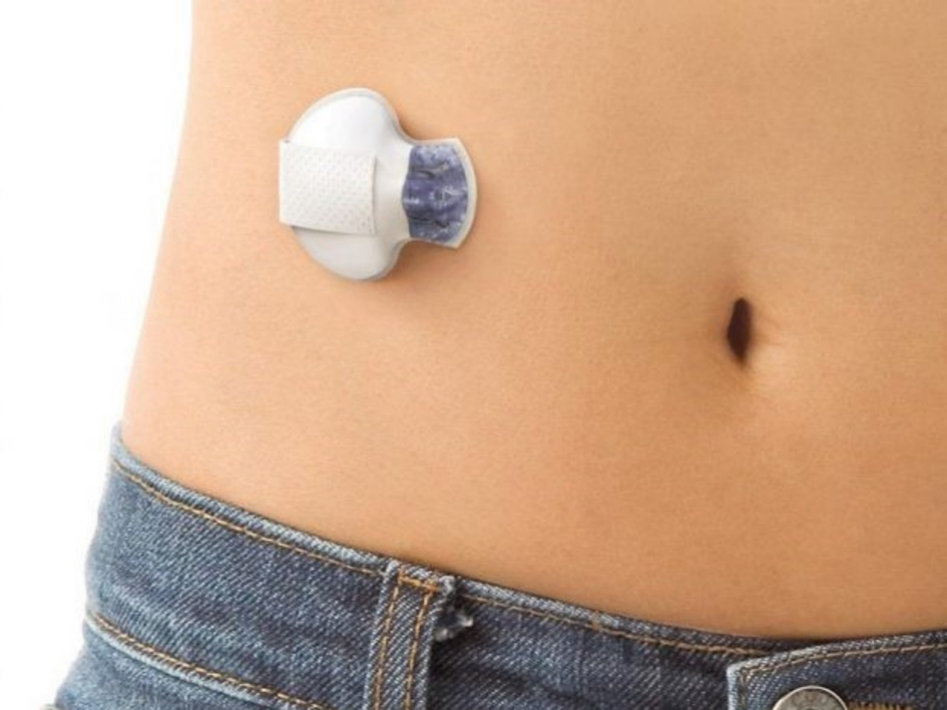
The Enlite glucose sensor with The Guardian 2 Link transmitter attached.

It connects to the glucose sensor and sends glucose data wirelessly to the MiniMed640G device (figure 2).

#### The MiniMed 640G device

**Figure 3:**
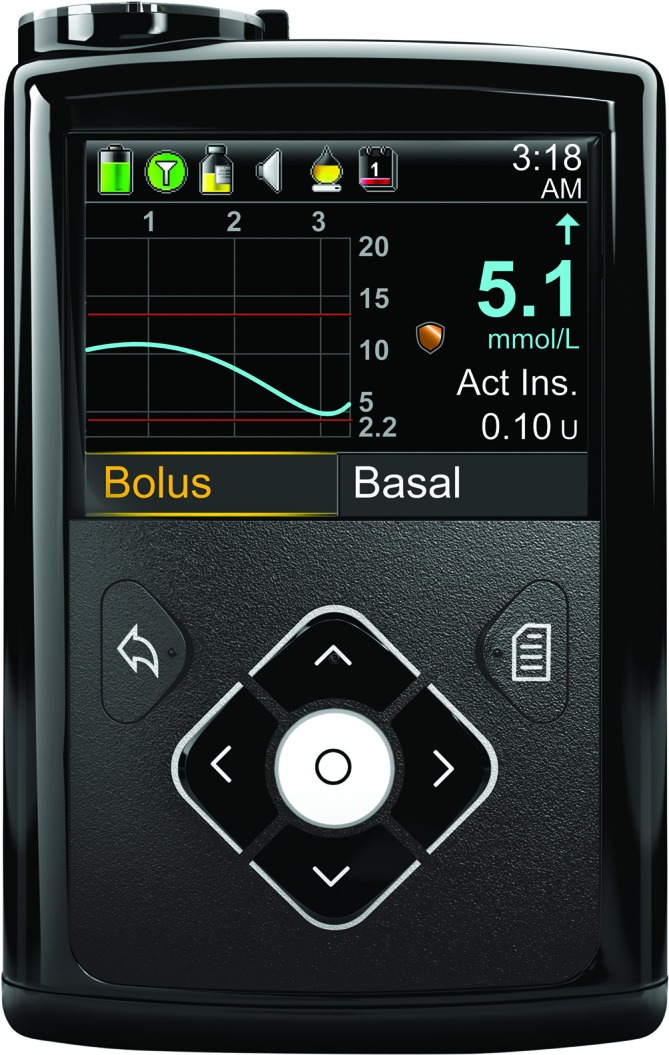
MiniMed 640G (Medtronic, Watford UK).

The 640G device as well as providing continuous glucose values will store data so that it can be analysed to track patterns and improve glucose management. Glucose data will be downloaded from the 640G at the end of the study period to a computer for analyses. This device received CE mark in 2014 (CE certificate No. 8857) (figure 3).

## Blinding

To ensure that the real-time data are not available to staff caring for babies in the control arm of the study the CGM will be kept secure in an opaque bag with tamper tag seals. The bags will be opened for calibration in every 12 hours, and each baby will have a log kept of timings of when the tamper tag is broken/resealed.

## Outcomes

The primary outcome is percentage of time SG in target range of 2.6–10 mmol/L. This was selected after consultation as it represents internationally, the clinically most widely accepted target range for glucose control for this population.

Secondary outcomes have been selected to provide further evidence around efficacy, acceptability and safety. These include for efficacy:

- Mean SG.
- Percentage of time SG in range of 4–8 mmol/L.
- SG variability within individuals as assessed by within patient SD.
- Percentage of time glucose levels in hyperglycaemic range, SG >15 mmol/L.

Acceptability will be assessed by a specifically designed staff questionnaire which will be completed anonymously on day 3 and 7 by the clinical team caring for the baby as well as a parent questionnaire on day 7.

Safety outcomes include both measures of BG and SG to address potential differences in methodologies and to provide data on prevalence of exposures that may be undetected clinically in the control arm of the study:

- Incidence of hypoglycaemia defined as any episode of BG >2.2 mmol/L and <2.6 mmol/L.
- Incidence of hypoglycaemia defined as continuous episode of CGM SG <2.6 mmol/L for >1 hour.
- Incidence of severe hypoglycaemia defined as any episode of BG ≤2.2 mmol/L.

Health economics: cost-effectiveness will be expressed in terms of incremental cost per additional case of adequate glucose control between 2.6 and 10 mmol/L.

## Sample size

Based on data from the REACT feasibility study and historical control data, we conservatively assume that the SD of the primary endpoint is 22%. A sample size of 200 participants will enable a treatment effect of a 10% increase in the mean value of the primary endpoint to be detected with 90% power using a two-sided 5% significance test in the primary analysis. Based on a consensus of expert opinion a difference of 10% is believed to be of minimal clinical relevance. It is expected that a small number of patients will be withdrawn from the study. Reasons for these withdrawals include transfer to participant’s local NICU, withdrawal of parental consent or death.

## Recruitment plan

All babies will be recruited within 24 hours of birth at one of the NICUs that has been approved for study participation. Due to the short time frame from birth to recruitment potentially eligible babies will be identified in a number of ways: (1) liaison with the obstetric team to highlight mothers at risk of preterm delivery, (2) liaison with the neonatal transport team to identify babies who have been born in local units being transferred to a study centre and (3) liaison with the NICU clinical team of study centres. Screening of eligible patients will be undertaken in collaboration with the clinical team and families approached only if considered eligible and the families consent to being approached about study involvement. Screening logs will be reviewed regularly by the coordinating centre to identify any issues around recruitment.

The REACT recruitment centres are level 3 NICUs. They have been selected either because of their previous experience of using CGM in the preterm infant, or they are centres with a proportionately large number of babies which would fulfil the study inclusion criteria and represent an international range of clinical practice and thus provide generalisability of the intervention. The first patient was recruited in July 2016, and the end of study is planned for November 2018.

## Randomisation

Randomisation will take place within 24 hours of delivery and babies will be randomised in a 1:1 ratio into control and intervention arms of the study using a central web randomisation system, TENALEA. The randomisation will use blocked stratified randomisation. The stratification factors will be to recruiting centres and gestation (<26 weeks gestation, ≥26 weeks gestation). The programme will notify the local research team of treatment allocation who will then inform their clinical team regarding the practicalities of management. This is an open study in which the clinical and research teams and parents will be aware of the study arm and intervention.

## Data management and analyses

Data collection will be undertaken from birth to 36 weeks corrected gestational age. If a baby has been discharged from their recruiting NICU, the research team will use local and national databases, local contacts and links with parents to ensure complete follow-up data is obtained. All data will be sent to the coordinating centre in Cambridge where it will be entered onto a MACRO database. All data will be collected, transferred and stored to comply with Good Clinical Practice (GCP) and Data Protection legislation. Access to data will only be granted to authorised personnel involved in study management or for auditing/monitoring to comply with regulations. To maintain high-quality standard of data entry, the data base will be tested and validated prior to use.

### Efficacy

This will be assessed by comparison of data collected by real-time CGM in the intervention arm and blinded CGM in the control infants.

### Clinical acceptability

Parents, nurses and medical staff, caring for babies in the study will be asked to complete study-specific questionnaires.

### Safety

This will be assessed in three areas: incidence of hypoglycaemia measured as part of clinical care (BG levels) and after review of SG data; device safety through adverse device effect (ADE) reporting; and acute mortality and morbidity outcomes as part of the case report form (CRF).

### Costs for economic evaluation

Data will be collected on the health service resources used in the treatment of infants during the period between randomisation and 36 weeks gestation and based on British Association of Perinatal Medicine standard criteria for level of care, as well as neonatal complications. Current UK unit costs will be applied to each resource item and a per diem cost for each level of neonatal care will be based on Department of Health reference costs calculated on a full absorption costing basis.

## Data analyses

The primary endpoint will be analysed using linear regression to estimate the absolute difference in time SG in target of 2.6–10 mmol/L, adjusting for baseline variables (centre, gestation). Analyses will be undertaken both for intention to treat and as treated populations. Estimates of treatment effect, with 95% CIs and p values will be provided. Secondary endpoints that are continuous variables will be analysed in a similar fashion. Secondary endpoints that are counts or binary variables will be analysed using an appropriate regression framework. Methods will be used to reduce the likelihood of a type I error. All of the efficacy endpoints will be ranked in order of importance: mean SG, percentage of time SG in target of 4–8 mmol/L, SG variability within individuals as assessed by within-patient SD, percentage of time glucose levels in hyperglycaemic range—SG >15 mmol/L.

Continuous variables will report the mean, median, SD, range, maximum and minimum. Binary or categorical endpoints will be represented using frequency tables in the ‘p% (r/n)’ format. The analysis will look for a treatment interaction effect with the following baseline variables: centre, sex, corrected gestational age, birthweight SDs, use of antenatal steroids, maternal chorioamnionitis and maternal diabetes using the regression framework in an exploratory, non-confirmatory manner.

An incremental cost-effectiveness analysis will be performed. In the baseline analysis, the economic evaluation will be expressed as the incremental cost per additional case of adequate glucose control. Adequate control will be considered as 80% of time in target. Given the multinational nature of the trial, the hierarchical structures of the cost and outcomes data will be taken into account in the analysis plan. Due to the known limitations of within-trial economic evaluations, we will also construct a decision-analytical model to model the cost-effectiveness of CGM beyond the time horizon of the trial.

## Site training

Research teams at each site will be required to have up-to-date GCP training and have undertaken training in study procedures including use of the CGM and Nova Biomedical point of care devices. Paper and online resources as well as a call line will be available to support the research teams.

## Monitoring

Data returns will be continually monitored by the central team for completeness and timeliness of all data returned. Compliance with intervention strategy in each study arm will also be reviewed to ensure there is not ‘crossover’ between study arms. A monitoring plan is in place determining frequency and scope of site monitoring based on continuing risk review. Face-to-face monitoring visits will initially be undertaken within the first 6 months and then adjusted following assessment of recruitment rate, number of data queries and serious adverse event (SAE) reports. The study sites will provide direct access to all trial-related source data and reports for the purpose of monitoring and auditing by the central study team, sponsor and regulatory authorities as required.

## Data and safety monitoring

The data and safety monitoring committee (DSMC) is responsible for safeguarding the interests of the trial participants and making recommendations to the trial steering committee (TSC). The REACT DSMC roles and responsibilities and operating procedures are defined in the REACT DSMC Charter. It is composed of three independent multidisciplinary experts who are not involved in the conduct of the trial in any way. They met prior to the initiation of enrolment and determined a plan to review the protocol, compliance, safety and AEs and outcome data after a prespecified number of babies have been recruited. The TSC is composed of five to six independent members and has a Charter defining the member’s roles and responsibilities. The TSC provide advice, through its chair, to the chief investigator and report to trial sponsor and trial funder.

Safety will be assessed continuously during each baby’s stay in NICU. The frequency of AE and SAE as defined by The International Conference on Harmonisation and that would normally require reporting within a clinical trial is anticipated to be high in the population being studied despite the low risk of the study intervention. Following discussions with the Medicines and Healthcare Products Regulatory Agency (MHRA) and in accordance with regulatory guidance which allows for exceptions in such circumstances a modified reporting plan was agreed.

Any ADE will be recorded and reported to the coordinating centre. All device deficiencies that might have led to a serious ADE (SADE) if suitable action had not been taken; intervention had not been made or if circumstances had been less fortunate, will be reported to the Sponsor as for SAEs/SADEs. AEs will be recorded in the notes and some will be captured as exploratory outcomes as part of the CRF. SAEs are common in this population, therefore, the MHRA requested the following expectation for safety reporting:

### During the intervention period of the study (study days 1–7)

The following expected SAEs will need to be recorded in the CRF (safety log) and reported using the safety report form to the sponsor within 24 hours of awareness of the event: (1) death, (2) culture positive infection, (3) severe hypoglycaemia (<2.6 mmol/L), (4) seizures, (5) any other related SAE.

### Postintervention period (study day 7 until end of study)

Important medical outcomes for the trial will be captured in the CRF at the 36 weeks corrected gestation assessment. Other SAEs are anticipated events for this study population and do not need to be recorded or reported separately as an SAE if judged by the clinical team to be unrelated to the study.

## Ethics and dissemination

The investigator or a suitably qualified person designated by the principal investigator will receive written informed consent from the patient’s parent/legally acceptable representative before any trial-specific activity is performed. Clinical trial authorisation has been granted by the MHRA (REF: CI/2016/0011). Written approvals will be received from individual hospital sites prior to recruitment. Approvals have also been obtained from Medical Ethics Review Committee, VU University Medical Centre, Amsterdam and Heath Care Inspectorate (REF: 2017–1 398434/VlO 14949), The Netherlands and the Research Ethics Committee, Sant Joan de Déu Research Foundation, Barcelona, and Ministry of Health Social Services and Equality (REF: 591/16/EC), Spain. The chief investigator will ensure that the trial is conducted in accordance with the principles of the Declaration of Helsinki and in conformity with the medical devices regulations and any relevant amendments. The findings of the trial will be prepared and presented at national and international meetings and conferences and published in peer-reviewed journals by the academic team.

## Patient and public involvement

Consultation with the parents from the local parent support group was undertaken to help inform trial design. Parents are being asked to provide feedback on study involvement as part of the protocol. The TSC includes a lay person. We will send newsletters to parents to update them on study progress.

## Conclusions

The REACT trial is an international multicentre trial which will randomise 200 preterm babies (≤1200 g and ≤24 hours of age) to receive either real-time CGM or standard clinical management of glucose control (with blinded CGM data collection). This study will determine if real-time CGM can support better targeting of glucose control in these babies reducing the risk of both hyperglycaemia and hypoglycaemia. This has the potential to impact on both the acute management, and in the future on outcomes of preterm babies who are at risk from glucose dysregulation.

## Supplementary Material

Reviewer comments

Author's manuscript
