# Supplementary material for: Protocol of a randomised controlled trial of real-time continuous glucose monitoring in neonatal intensive care ‘REACT’
Source: BMJ Open. 2018 Jun 4;8(6):e020816. doi: 10.1136/bmjopen-2017-020816 (PMC5988133; doi:10.1136/bmjopen-2017-020816)
Supplement: Supplementary file 1 [file bmjopen-2017-020816supp001.pdf]

Appendices

Appendix 1: Paper algorithm for use in intervention arm only

Sensor Glucose mmol/l	Falling	Stable	Rising
<2.6	**Check Blood Glucose**<br>Stop any Insulin & Check all lines<br>Give additional Dextrose<br>Consider starting 20% Dextrose at 1ml/kg/hr	**Check Blood Glucose**<br>Stop any Insulin & Check all lines<br>Give additional Dextrose<br>Consider starting 20% Dextrose at 1ml/kg/hr	**Check Blood Glucose**<br>Review infusions & check lines<br>Ensure Insulin is not running<br>Consider starting/increasing<br>20% Dextrose at 1ml/kg/hr
**2.6-4.0**	**Check Blood Glucose**<br>Stop any Insulin & Check all lines<br>Give additional Dextrose<br>Consider starting 20% Dextrose at 1ml/kg/hr	**Check Blood Glucose**<br>Stop any Insulin & Check all lines<br>Give additional Dextrose<br>Consider starting 20% Dextrose at 1ml/kg/hr	**Observe the rate of rise**<br>Review infusions & check lines<br>Ensure Insulin is not running<br>Consider need for additional Dextrose
Target Range<br>4.0 - 8.0	**IN TARGET**<br>If the rate of fall means you will be <4.0mmol/l<br>within 1 hour consider reducing Insulin	**IN TARGET**	**IN TARGET**<br>Consider weaning any additional<br>20% Dextrose
**8.0-10.0**	**Observe the rate of fall**<br>Consider ***reducing*** Insulin infusion rate by 25%	Stop any additional 20% Dextrose<br>or<br>Start Insulin at 0.05 units/kg/hr<br>or<br>if Insulin is already running ***increase***<br>Insulin infusion rate by 50%	Stop any additional 20% Dextrose<br>or<br>Start Insulin at 0.05 units/kg/hr<br>or<br>if Insulin is already running ***increase***<br>Insulin infusion rate by 50%
**10-15.0**	**Observe the rate of fall**<br>Consider ***increasing*** Insulin infusion rate by 25%	Stop any additional 20% Dextrose<br>or<br>Start Insulin at 0.05 units/kg/hr<br>or<br>if Insulin is already running ***increase***<br>Insulin infusion rate by 50%	Stop any additional 20% Dextrose<br>or<br>Start Insulin at 0.05 units/kg/hr<br>or<br>if Insulin is already running ***increase***<br>Insulin infusion rate by 50%
**> 15**	**Observe the rate of fall**<br>Consider ***increasing*** Insulin infusion rate by 50%	Start Insulin at 0.05 units/kg/hr<br>**or**<br>consider ***increasing*** Insulin infusion rate<br>by 100% (that is: Double)<br>**Always check infusion lines if there is little or no response to an intervention**	Start Insulin at 0.05 units/kg/hr<br>**or**<br>consider ***increasing*** Insulin infusion rate<br>by 100% (that is: Double)<br>**Always check infusion lines if there is little or no response to an intervention**

CRITICAL

CONCERN

IN TARGET

Please remember continuous glucose sensor readings are provided to support clinical management.
They provide additional information on trends in glucose levels which should be used to guide the need for blood glucose measurement.
Capillary/venous blood glucose levels are more accurate.
Always check infusion lines if there is little or no response to an intervention
