# Supplementary material for: Protocol of a randomised controlled trial of real-time continuous glucose monitoring in neonatal intensive care ‘REACT’
Source: BMJ Open. 2018 Jun 4;8(6):e020816. doi: 10.1136/bmjopen-2017-020816 (PMC5988133; doi:10.1136/bmjopen-2017-020816)
Supplement: Author's manuscript [file bmjopen-2017-020816.draft_revisions.pdf]

BMJ Open is committed to open peer review. As part of this commitment we make the peer review history of every article we publish publicly available.

When an article is published we post the peer reviewers' comments and the authors' responses online. We also post the versions of the paper that were used during peer review. These are the versions that the peer review comments apply to.

The versions of the paper that follow are the versions that were submitted during the peer review process. They are not the versions of record or the final published versions. They should not be cited or distributed as the published version of this manuscript.

BMJ Open is an open access journal and the full, final, typeset and author-corrected version of record of the manuscript is available on our site with no access controls, subscription charges or pay-per-view fees (http://bmjopen.bmj.com).

If you have any questions on BMJ Open's open peer review process please email info.bmjopen@bmj.com

BMJ Open

Protocol of a Randomised Controlled Trial of Real Time Continuous Glucose Monitoring in Neonatal Intensive Care

Journal:	*BMJ Open*
Manuscript ID	bmjopen-2017-020816
Article Type:	Protocol
Date Submitted by the Author:	02-Dec-2017
Complete List of Authors:	Beardsall, Kathryn; University of Cambridge Department of Paediatrics<br>Thomson, Lynn; University of Cambridge, Department of Paediatrics<br>Guy, Catherine; University of Cambridge, Department of Paediatrics<br>Weissenbruch, Mirjam; VU University Medical Center, P.O box 7057, 1007 MB Amsterdam, NL<br>Iglesias, Isabel; Hospital Sant Joan de Deu<br>Muthukumar, Priya; Norfolk & Norwich University Hospitals NHS Foundation Trust<br>Somisetty, Sateesh; Luton & Dunstable University Hospital<br>Bond, Simon; Cambridge University Hospitals NHS Foundation Trust, Cambridge clinical trials unit<br>Petrou, Stavros; University of Warwick, Warwick Medical School<br>Dunger, David; University of Cambridge, Paediatrics
Keywords:	Randomised Control Trial, Glucose, Continuous monitoring, Hyperglycaemia, Hypoglycaemia

SCHOLARONE™ Manuscripts

only

Protocol of a Randomised Controlled Trial of Real Time Continuous Glucose Monitoring in Neonatal Intensive Care

REACT

Kathryn Beardsall MD FRCPCH,[1,2] Lynn Thomson RSCN,[1,2] Catherine Guy,[1] Mirjam M van Weissenbruch MD PhD,[3] Isabel Iglesias MD,[4] Priya Muthukumar MRCPCH,[5] Sateesh Kumar Somisetty MRCPCH, [6] Simon Bond,[7] Stavros Petrou[8] and David Dunger FAcad Med Sci[1]

Author Affiliations
1. University Department of Paediatrics, University of Cambridge, Box 116, Cambridge Biomedical Campus, Cambridge, CB2 0QQ, UK. 2. Cambridge University Hospitals NHS Foundation Trust, Hills Road, Cambridge, CB2 2QQ, UK. 3. VU University Medical Center, P.O box 7057, 1007 MB Amsterdam, NL 4. Sant Joan de Déu, 08950 Esplugues de Llobregat, Barcelona, Spain. 5. Norfolk & Norwich University Hospitals NHS Foundation Trust, Colney Lane, Norwich, NR4 7UY, UK. 6. Luton & Dunstable University Hospital, Lewsey Road, Luton LU4 0DZ, UK. 7. Cambridge Clinical Trials Unit, Cambridge University Hospitals NHS Foundation Trust, Cambridge, CB2 0QQ, UK. 8. Division of Health Sciences, Warwick Medical School, University of Warwick, Coventry, CV4 7AL, UK

Addresss for Correspondence:
Dr K Beardsall email: kb274@cam.ac.uk 01223 746791

Short Title: RCT of Real time CGM in NICU

Key words:
randomised controlled trial, glucose, continuous monitoring, hyperglycaemia, hypoglycaemia

Abbreviations
BG blood glucose, CGM continuous glucose monitoring, DMC Data monitoring committee, NICU neonatal intensive care unit, SG Sensor glucose, TSC Trial Steering committee

For peer review only

The Corresponding Author has the right to grant on behalf of all authors and does grant on behalf of all authors, a worldwide licence to the Publishers and its licensees in perpetuity, in all forms, formats and media (whether known now or created in the future), to i) publish, reproduce, distribute, display and store the Contribution, ii) translate the Contribution into other languages, create adaptations, reprints, include within collections and create summaries, extracts and/or, abstracts of the Contribution, iii) create any other derivative work(s) based on the Contribution, iv) to exploit all subsidiary rights in the Contribution, v) the inclusion of electronic links from the Contribution to third party material where-ever it may be located; and, vi) licence any third party to do any or all of the above."

"All authors have completed the ICMJE uniform disclosure form at www.icmje.org/coi_disclosure.pdf and declare: KB on behalf of the research team has received NIHR EME grant and provision of equipment from Medtronic and Nova Biomed to support this trial. There are no other relationships or activities that could appear to have influenced the submitted work."

For peer review only

Abstract

Introduction: Hyperglycaemia is common in the very preterm infant and has been associated with adverse outcomes. Preventing hyperglycaemia without increasing the risk of hypoglycaemia has proved challenging. The development of real time continuous glucose monitors (CGM) to inform treatment decisions provides an opportunity to reduce this risk. This study aims to assess the feasibility of CGM combined with a specifically designed paper guideline to target glucose control in the preterm infant.

Methods and Analyses: The REACT Randomised controlled Trial (Real Time Continuous Glucose Monitoring in Neonatal Intensive Care) is an international multicentre randomised controlled trial. Two hundred preterm infants ≤1200g, and ≤24 hours of age will be randomly allocated to either real time continuous glucose monitoring, or standard care (with blinded CGM data collection). The primary outcome is time in target 2.6-10mmol/l during the study intervention assessed using continuous glucose monitoring. Secondary outcomes include efficacy relating to glucose control, utility including staff acceptability, safety outcomes relating to incidence and prevalence of hypoglycaemia and health economic analyses.

Ethics and Dissemination: The REACT Trial has been approved by the NHS Health Research Authority National Research Ethics Service Committee East of England (Cambridge Central); Medical Ethics Review Committee, VU University Medical Centre, Amsterdam, The Netherlands and the Research Ethics Committee, Sant Joan de Déu Research Foundation, Barcelona, Spain. Recruitment began in July 2016 and will continue until mid 2018. The Trial has been adopted by the National Institute of Health Research Clinical Research Network portfolio (ID: 18826) and is registered with the ISRCTN registry (ID: 12793535). Dissemination plans include presentations at scientific conferences, scientific publications and efforts at stakeholder engagement.

For peer review only

For peer review only - http://bmjopen.bmj.com/site/about/guidelines.xhtml

Strengths and Limitations

The comparison of real time CGM data with blinded CGM data in the control study arm will provide detailed comparable data on efficacy and safety between study arms.

As an International multicentre trial the results will be generalizable across a range of neonatal intensive care settings.

Input by staff and parents within the trial itself as well as part of the Trial management will provide information on utility and facilitate translation of the outcomes into clinical practice.

The study requires recruitment within 24 hours of preterm birth, which requires a significant commitment from the clinical and research teams if it is to be successful.

The study is powered to detect a difference in the Primary outcome 'Time in target' (2.6-10mmol/l), but will not have the power to detect the impact on clinical outcomes.

For peer review only

Introduction

In utero, glucose levels are normally maintained between 4-6mmol/l [1], but infants born preterm are at risk of both hyperglycaemia and hypoglycaemia[2]. Hyperglycaemia and hypoglycaemia have both been associated with increased mortality and morbidity of preterm babies[3,4]. Hyperglycaemia can lead to acute problems of a persistent osmotic diuresis and metabolic acidosis which can be difficult to control and has been associated with increased risk of intraventricular haemorrhage and patent ductus arteriousus[5]. Hyperglycaemia has also been associated with increased long term morbidity, including increased risk of retinopathy of prematurity[4 6-8]. Hypoglycaemia is associated with characteristic occipital temporal lesions[8]. Attempts to reduce risks associated with hyperglycaemia in adult and paediatric and neonatal intensive care (NICU) have resulted in increased risk of hypoglycaemia[9 10 11]. This is of particular concern for the very preterm infant in whom there is very varied insulin sensitivity, which increases the risk of hypoglycaemia. In addition the developing brain appears to be particularly vulnerable to both hyperglycaemic [12], and hypoglycaemic insults. Early postnatal glucose control may be an important modifiable risk factor for clinical outcomes. A recent Cochrane review has highlighted the need for further studies into the impact of interventions to improve glucose control in these infants[13].

Managing glucose control is dependent in part on methods of measuring and monitoring glucose levels. Within NICU glucose measurements are currently limited to intermittent blood sampling [14], with long periods when glucose levels are unknown. In contrast, other physiological parameters such as oxygen saturation, blood pressure and heart rate are all monitored continuously to prevent wide fluctuations. It is increasingly thought that fluctuations in glucose levels may also have a significant impact on long term outcomes [15]. The reason for the intermittent measurement is that current methodologies rely on blood sampling either from a central arterial line or by heel prick. Clinical care for preterm infants aims to reduce the frequency of handling [16] and volume of blood sampled as this has been shown to improve outcomes.

Developments in the measurement of glucose levels in patients with diabetes mellitus include continuous glucose monitoring of interstitial glucose levels [17]. Real time data on interstitial glucose levels now provides information on glucose trends with the potential for earlier intervention and prevention of both hyperglycaemia and hypoglycaemia. Benefits within adult intensive care remain controversial [18], however the benefits in the setting of NICU may be more marked as blood glucose (BG) measurements are taken much less frequently in these small babies. There have also been key developments in the technology including extended life of sensors (previously 72 hours) and improved accuracy [19]. The latter is particularly relevant due to the threshold levels of hypoglycaemia and hyperglycaemia (<2.6 and >10mmol/L) which are more extreme than in adults and are at the limits of accuracy of many methods of glucose measurement.

Blinded CGM has been used in preterm babies within clinical trials[2 20 21], and studies of real time devices have shown a benefit in providing early warning of and prevention of hypoglycaemia [22]. Studies have not so far attempted to use CGM to guide clinical management to support the targeting of glucose control in preterm infants. A single centre feasibility study of the real time monitors demonstrated that sensor glucose (SG) values are comparable with BG values (REACT feasibility study REC Ref: 14/EE/0127). Data from the feasibility has helped to design this randomised controlled trial. This multicentre randomised controlled trial will determine whether real time continuous glucose monitoring, with support from a paper algorithm, can help improve the management of glucose control in terms of efficacy, safety and clinical acceptability. This will not only enhance the short term management of glucose control in infants requiring intensive care but by reducing the risks associated with both hyperglycaemia and hypoglycaemia may impact on long term clinical outcomes.

Trial Objectives

The REACT Trial will evaluate efficacy, safety, utility and cost effectiveness of real time continuous glucose monitoring (CGM) in preterm infants in NICU. Our primary hypothesis is that the use of real time CGM will improve the time a baby's glucose levels (measured using CGM)

For peer review only - http://bmjopen.bmj.com/site/about/guidelines.xhtml

remain within the target 2.6-10mmol/l (widely accepted clinical target for glucose control), compared to standard clinical practice (with blinded CGM data collection). Secondary objectives include evaluation of clinical acceptability in the preterm infant (using staff and parent questionnaires) and safety in relation to the device itself and risk of hypoglycaemia.

Methods

Study Design

This is a multicentre interventional, randomised controlled trial of CGM compared to standard clinical management (control). We will recruit potential participants within 24 hours of birth and continually monitor their glucose levels for 6 days. Data will be collected until 36 weeks corrected gestational age. No other aspects of concomitant care are prohibited during the trial.

Eligibility Criteria

Inclusion Criteria

Infants who have a birth weight ≤ 1200g, are ≤ 24 hours of age, ≤ 33+6 weeks gestation and in whom written informed parental consent has been received.

Exclusion Criteria

Any lethal congenital abnormality known at trial entry, any congenital metabolic disorder known at trial entry and any neonates who, in the opinion of the treating clinician at trial entry, have no realistic prospect of survival.

Intervention

Babies will be randomised in a 1:1 ratio, into control and intervention arms of the study using a web randomisation system, TENALEA. This is an open study in which the clinical staff, research team and parents will be aware of the study arm and intervention. All babies will have a subcutaneous sensor inserted, Enlite™(Medtronic) that will be linked to a Medtronic MiniMed 640G™ System and will be calibrated with point of care BG levels. For consistency across sites calibration will be standardized by providing all units with Nova StatStrip™ meters.

Standard care with blinded CGM data collection (Control)

These infants will have their glucose control monitored and managed according to standard clinical practice using intermittently sampled BG levels. Nutritional delivery (including glucose) and insulin delivery will be prescribed according to the standard clinical guidelines within each unit. The CGM device will collect glucose data continuously but the clinical team will be blinded to the data as the monitor will be kept covered and fastened with a tamper proof seal.

Real Time Continuous Glucose Monitoring Device with paper algorithm (Intervention)

The CGM data will be open to view by the clinical team during the first week of the baby's life and the staff will be advised to read and record the SG data hourly as part of standard clinical monitoring. This will support staff to use the additional data available from real time monitoring to guide timing of BG measurement and changes in clinical management. Interventions to target glucose control will then be guided by the specifically designed paper algorithm. This algorithm was developed during the REACT feasibility study (REC Ref: 14/EE/0127).

Medical Devices: MiniMed 640G System

The MiniMed 640G System is indicated for glucose monitoring and for continuous delivery of insulin, for the management of diabetes mellitus in persons requiring insulin. Monitoring equipment only will be used for this study. The system being used comprises linking the Enlite sensor

For peer review only

(Medtronic, Northridge, CA, USA) using the Guardian 2 Link transmitter to the MiniMed 640G which then displays the glucose data in real time.

i. Sensor

The Enlite sensor (Medtronic, Northridge, CA, USA) is a CGM sensor which received CE mark in 2013 (CE certificate No. 21024). The sensor (figure 1) comprises a disposable subcutaneous oxidase-based platinum electrode that catalyses interstitial glucose generating an electrical current every 10 seconds which is transmitted to a monitor for display and/or recording. The data will be recorded and/or displayed as an averaged value every 5 minutes, giving a total of 288 readings per day. Glucose values outside the range 2.2-24.0 mmol/l (40-430 mg/dl) are reported as < 2.2 mmol/l (40 mg/dl), or >24 mmol/l (430 mg/dl) respectively.

Figure 1: Enlite sensor

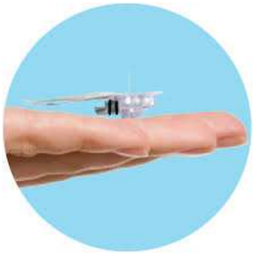

The sensor will be inserted subcutaneously (into the thigh) by hand, not using the standard insertion device, thus ensuring the sensor is inserted into the subcutaneous tissue. The sensors are soft and flexible, approximately 8.75mm in length and are mounted inside a hollow needle to allow for subcutaneous insertion. Once the sensor is inserted the introducer needle will be withdrawn, and the sensor attached to a small Guardian™ 2 Link transmitter (CE Mark 2013; Certificate No. 8858) for data transfer to the MiniMed 640G System for data viewing. The sensor will then be secured with a clear occlusive dressing (again trimmed to ensure minimal contact with the infant's skin), so that the insertion site can be inspected daily. A blood sample will be required every 12 hours to ensure calibration of the sensor. Sensors will be removed after 7 days.

Figure 2: The Enlite sensor with Guardian 2 Link transmitter attached

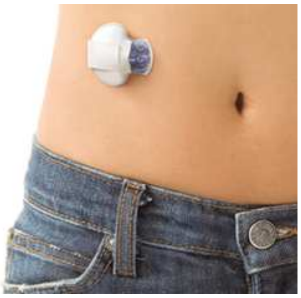

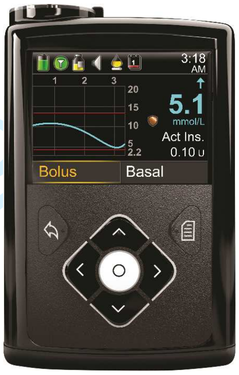

Figure 3: MiniMed 640G monitor

ii. ***Guardian 2 Link Transmitter*** (Figure 2)

It connects to the glucose sensor and sends glucose data wirelessly to the MiniMed® 640G monitor (pump).

iii. ***The MiniMed 640G/monitor (pump)*** (Figure 3)

The 640G monitor as well as providing continuous glucose values will store data so that it can be analysed to track patterns and improve glucose management. Glucose data will be downloaded from the 640G at the end of the study period to a computer for analyses. This device received CE mark in 2014 (CE certificate No. 8857).

For peer review only - http://bmjopen.bmj.com/site/about/guidelines.xhtml

Blinding

To ensure that the real time data is not available to staff caring for babies in the control arm of the study the CGM will be kept secure in an opaque bag with tamper tag seals. The bags will be opened for calibration every 12 hours, and each baby will have a log kept of timings of when the tamper tag is broken/resealed.

Outcomes

The *Primary outcome* is percentage of time SG in target range of 2.6-10mmol/l. This was selected after consultation as it represents internationally, the clinically most widely accepted target range for glucose control for this population.
Secondary Outcomes have been selected to provide further evidence around efficacy, acceptability and safety. These include for efficacy:

- mean SG
- percentage of time SG in range of 4-8mmol/l
- SG variability within individuals as assessed by within patient standard deviation
- percentage of time glucose levels in hyperglycaemic range, SG >15mmol/l.

Acceptability will be assessed by a specifically designed staff questionnaire which will be completed anonymously on day 3 and 7 by the clinical team caring for the baby as well as a parent questionnaire on day 7.

Safety outcomes include both measures of BG and SG to address potential differences in methodologies and to provide data on prevalence of exposures that may be undetected clinically in the control arm of the study:

- Incidence of hypoglycaemia defined as any episode of BG >2.2mmol/l and <2.6mmol/l
- Incidence of hypoglycaemia defined as continuous episode of CGM SG <2.6mmol/l for >1hour
- Incidence of severe hypoglycaemia defined as any episode of BG ≤2.2mmol/l

Health Economics: cost-effectiveness will be expressed in terms of incremental cost per additional case of adequate glucose control between 2.6mmol/l – 10mmol/l

Sample Size

Based on data from the REACT feasibility study and historical control data, we conservatively assume that the SD of the primary endpoint is 22%. A sample size of 200 participants will enable a treatment effect of a 10% increase in the mean value of the primary endpoint to be detected with 90% power using a two-sided 5% significance test in the primary analysis. Based on a consensus of expert opinion a difference of 10% is believed to be of minimal clinical relevance. It is expected that a small number of patients will be withdrawn from the study. Reasons for these withdrawals include transfer to participant's local NICU, withdrawal of parental consent or death.

Recruitment Plan

All babies will be recruited within 24 hours of birth at one of the NICUs that has been approved for study participation. Due to the short time frame from birth to recruitment potentially eligible babies will be identified in a number of ways: i. liaison with the obstetric team to highlight mothers at risk of preterm delivery, ii. Liaison with the neonatal transport team to identify babies who have been born in local units being transferred to a study centre, iii. Liaison with the NICU clinical team of study centres. Screening of eligible patients will be undertaken in collaboration with the clinical team and families approached only if considered eligible and the families consent to being approached about study involvement. Screening logs will be reviewed regularly by the coordinating centre to identify any issues around recruitment.

For peer review only - http://bmjopen.bmj.com/site/about/guidelines.xhtml

The REACT recruitment centres are level 3 NICUs. They have been selected either because of their previous experience of using continuous glucose monitoring in the preterm infant, or they are centres with a proportionately large number of babies which would fulfil the study inclusion criteria and represent an International range of clinical practice and thus provide generalisability of the intervention. The first patient was recruited in July 2016, and the end of study is planned for November 2018.

Randomisation

Randomisation will take place within 24 hours of delivery and babies will be randomised in a 1:1 ratio into control and intervention arms of the study using a central web randomisation system, TENALEA. The randomisation will use blocked stratified randomisation. The stratification factors will be to recruiting centres and gestation (<26 weeks gestation, ≥26 weeks gestation). The programme will notify the local research team of treatment allocation who will then inform their clinical team regarding the practicalities of management. This is an open study in which the clinical and research teams and parents will be aware of the study arm and intervention.

Data management and analyses

Data collection will be undertaken from birth to 36 weeks corrected gestational age. If a baby has been discharged from their recruiting NICU the research team will use local and national databases, local contacts and links with parents to ensure complete follow up data is obtained. All data will be sent to the coordinating centre in Cambridge where it will be entered onto a MACRO database. All data will be collected, transferred and stored to comply with GCP and Data Protection legislation. Access to data will only be granted to authorised personnel involved in study management or for auditing/monitoring to comply with regulations. To maintain high quality standard of data entry the data base will be tested and validated prior to use.

Efficacy: This will be assessed by comparison of data collected by real time CGM in the intervention arm and blinded CGM in the control infants.
Clinical Acceptability: Parents, nurses and medical staff, caring for babies in the study will be asked to complete study specific questionnaires.
Safety: This will be assessed in 3 areas: incidence of hypoglycaemia measured as part of clinical care (BG levels) and after review of SG data; device safety through adverse device effect reporting; and acute mortality and morbidity outcomes as part of the CRF.
Costs for Economic Evaluation: Data will be collected on the health service resources used in the treatment of infants during the period between randomisation and 36 weeks gestation and based on British Association of Perinatal Medicine standard criteria for level of care, as well as neonatal complications. Current UK unit costs will be applied to each resource item and a *per diem* cost for each level of neonatal care will be based on Department of Health reference costs calculated on a full absorption costing basis.

Data Analyses

The primary endpoint will be analysed using linear regression to estimate the absolute difference in time SG in target of 2.6 - 10mmol/l, adjusting for baseline variables (centre, gestation). Analyses will be undertaken both for intention to treat and as treated populations. Estimates of treatment effect, with 95% confidence intervals and p-values will be provided. Secondary endpoints that are continuous variables will be analysed in a similar fashion. Secondary endpoints that are counts or binary variables will be analysed using an appropriate regression framework. Methods will be used to reduce the likelihood of a type I error. All of the efficacy endpoints will be ranked in order of importance: mean SG, percentage of time SG in target of 4-8mmol/l, SG variability within individuals as assessed by within-patient standard deviation, percentage of time glucose levels in hyperglycaemic range - SG >15mmol/l.

Continuous variables will report the mean, median, SD, range, max and min. Binary or categorical endpoints will be represented using frequency tables in the "p% (r/n)" format. The analysis will look for a treatment interaction effect with the following baseline variables: centre, sex, corrected

For peer review only

gestational age, birth weight SDS, use of antenatal steroids, maternal chorioamnionitis and maternal diabetes using the regression framework in an exploratory, non-confirmatory manner.

An incremental cost-effectiveness analysis will be performed. In the baseline analysis, the economic evaluation will be expressed as the incremental cost per additional case of adequate glucose control. Adequate control will be considered as 80% of time in target. Given the multinational nature of the trial, the hierarchical structures of the cost and outcomes data will be taken into account in the analysis plan. Due to the known limitations of within-trial economic evaluations we will also construct a decision-analytical model to model the cost-effectiveness of CGM beyond the time horizon of the trial.

Site Training

Research teams at each site will be required to have up to date GCP training and have undertaken training in study procedures including use of the CGM and Nova Biomedical point of care devices. Paper and online resources as well as a call line will be available to support the research teams.

Monitoring

Data returns will be continually monitored by the central team for completeness and timeliness of all data returned. Compliance with intervention strategy in each study arm will also be reviewed to ensure there is not 'crossover' between study arms. A monitoring plan is in place determining frequency and scope of site monitoring based on continuing risk review. Face-to-face monitoring visits will initially be undertaken within the first 6 months and then adjusted following assessment of recruitment rate, number of data queries and SAE reports. The study sites will provide direct access to all trial related source data and reports for the purpose of monitoring and auditing by the central study team, Sponsor and regulatory authorities as required.

Data and safety monitoring

The Data and Safety Monitoring Committee (DMC) is responsible for safeguarding the interests of the trial participants and making recommendations to the Trial Steering Committee (TSC). The REACT DMC roles and responsibilities and operating procedures are defined in the REACT DMC Charter. It is composed of a 3 independent multidisciplinary experts who are not involved in the conduct of the trial in any way. They met prior to the initiation of enrolment and determined a plan to review the protocol, compliance, safety and adverse events and outcome data after a pre-specified number of babies have been recruited. The TSC is comprised of 5-6 independent members and has a Charter defining the member's roles and responsibilities. The TSC provide advice, through its Chair, to the Chief Investigator, and report to Trial Sponsor and Trial Funder.

Safety will be assessed continuously during each baby's stay in NICU. The frequency of Adverse Event (AE) and Serious Adverse Event (SAE) as defined by The International Conference on Harmonisation and that would normally require reporting within a clinical trial is anticipated to be high in the population being studied despite the low risk of the study intervention. Following discussions with the Medicines and Healthcare Products Regulatory Agency (MHRA) and in accordance with regulatory guidance which allows for exceptions in such circumstances a modified reporting plan was agreed.

Any Adverse Device Effect (ADE) will be recorded and reported to the coordinating centre. All device deficiencies that might have led to a Serious Adverse Device Effect (SADE) if suitable action had not been taken; intervention had not been made; or if circumstances had been less fortunate, will be reported to the Sponsor as for SAEs/SADEs. Adverse events will be recorded in the notes and some will be captured as exploratory outcomes as part of the CRF. SAEs are common in this population, therefore the MHRA requested the following expectation for safety reporting:

During the Intervention period of the study (study days 1-7)

For peer review only

The following expected SAEs will need to be recorded in the CRF (safety log) and reported using the safety report form to the sponsor within 24 hours of awareness of the event: i. Death, ii. Culture positive infection, iii. Severe hypoglycaemia (<2.6mmol/l), iv. Seizures, v. any other related SAE.

Post intervention period (study day 7 until end of study)

Important medical outcomes for the trial will be captured in the CRF at the 36 weeks corrected gestation assessment. Other SAEs are anticipated events for this study population and do not need to be recorded or reported separately as an SAE if judged by the clinical team to be unrelated to the study.

Ethics and Dissemination

The investigator or a suitably qualified person designated by the principal investigator will receive written informed consent from the patient's parent/legally acceptable representative before any trial-specific activity is performed. The Trial Protocol (version 4, 7 Nov 2016) has been approved by the NHS Health Research Authority National Research Ethics Service Committee East of England – Cambridge Central (REC REF 15/EE/0158). Clinical Trial authorisation has been granted by the MHRA (REF: CI/2016/0011). Written approvals will be received from individual hospital sites prior to recruitment. Approvals have also been obtained from Medical Ethics Review Committee, VU University Medical Centre, Amsterdam and Heath Care Inspectorate (REF: 2017-1 398434/VIO 14949), The Netherlands and the Research Ethics Committee, Sant Joan de Déu Research Foundation, Barcelona, and Ministry of Health Social Services and Equality (REF: 591/16/EC), Spain. The Chief Investigator will ensure that the trial is conducted in accordance with the principles of the Declaration of Helsinki and in conformity with the Medical Devices Regulations and any relevant amendments. The findings of the Trial will be prepared and presented at national and International meetings and conferences and published in peer reviewed journals by the academic team

Patient and Public Involvement

Consultation with the parents from the local parent support group was undertaken to help inform trial design. Parents are being asked to provide feedback on study involvement as part of the protocol. The TSC includes a lay person. We will send newsletters to parents to update them on study progress.

Conclusions

The REACT trial is an international multicentre trial which will randomise 200 preterm babies (≤1200g and ≤24 hours of age) to receive either real time continuous glucose monitoring or standard clinical management of glucose control (with blinded CGM data collection). This study will determine if real time CGM can support better targeting of glucose control in these babies reducing the risk of both hyperglycaemia and hypoglycaemia. This has the potential to impact on both the acute management, but also in the future on outcomes of preterm babies who are at risk from glucose dysregulation.

For peer review only

Acknowledgements

This project is funded by the Efficacy and Mechanism Evaluation (EME) Programme, an MRC and NIHR partnership and supported in terms of statistical support and data management by the Cambridge Clinical Trials Unit.

The authors are thankful for the support of the Cambridge NICU team who helped to shape the RCT design based on feedback from the feasibility study and members of the Department of Paediatrics in Cambridge for support with trial set up. They also wish to thank all the families that are currently and will be taking part in the study. It is also important to acknowledge all the members of the clinical teams who help to identify potentially eligible babies but moreover take the time and effort within a busy clinical environment to take on the additional engagement with the practicalities of a new study and new way of caring for babies. We also need to acknowledge the independent members of the TSC and DMEC who helped to finalise the protocol. REACT is a portfolio adopted study and receives support from the UK Children Research Network.

Contributors

The trial was conceptualised by KB and DBD with input from LT. II, PM, SS, SP, MW, SB are named Investigators on the REACT Trial. All authors contributed to design of the trial with specific additional contributions from each co-author within their area of expertise. KB is the lead doctor for the trial and prepared the first version and subsequent revisions of the manuscript. LT is the lead nurse for the trial and CG trial coordinator. All authors contributed to the manuscript and have approved the final manuscript prior to submission.

Collaborators/Study Investigators

REACT Investigators: Professor David Dunger, Dr Roman Hovorka, Dr Sateesh Somisetty, Dr Priya Muthukumar, Dr Mirjiam Weissenbruch, Dr Amanda Ogilvy Stuart and Dr Isabel Iglesias Platas.

Funding

The study is funded by NIHR: Evaluation, Trials and Studies Programme. Further support was provided by Medtronic for supplying MiniMed 640G System, Enlite sensors and Guardian 2 Link transmitters and Nova Biomedical provided all point of care equipment and training for glucose, lactate and ketone measurement. The trial is jointly sponsored by Cambridge University Hospitals NHS Foundation Trust and University of Cambridge.

Disclaimer

The Trial Sponsor and funders (NIHR and Medtronic) had no role in trial design, and will have no role in gathering of data, access to data, preparation of the manuscript or decision to publish the results. The views expressed in this publication are those of the author(s) and not necessarily those of the MRC, NHS, NIHR or the Department of Health.

The EME Programme is funded by the MRC and NIHR, with contributions from the CSO in Scotland and NISCHR in Wales and the HSC R&D Division, Public Health Agency in Northern Ireland.

Competing Interests

Dr Bond reports grants from NIHR, during the conduct of the study.

Ethics Approval

The Trial has been approved by the NHS Health Research Authority National Research Ethics Service Committee East of England – Cambridge Central (REC REF 15/EE/0158).

Provenance and Peer review

Not commissioned; externally peer reviewed

Open Access

For peer review only

This is an open access article distributed in accordance with the terms of the Creative Commons Attribution (CC BY 4.0) license, which permits others to distribute, remix adapt and build upon this work, for commercial use, provided the original work is properly cited.

For peer review only

References

1. Economides DL, Nicolaides KH, Campbell S. Metabolic and endocrine findings in appropriate and small for gestational age fetuses. *J Perinat Med* 1991;19(1-2):97-105.
2. Beardsall K, Vanhaesebrouck S, Ogilvy-Stuart AL, et al. Prevalence and determinants of hyperglycemia in very low birth weight infants: cohort analyses of the NIRTURE study. *J Pediatr* 2010;157(5):715-9 e1-3. doi: 10.1016/j.jpeds.2010.04.032
3. Sinclair JC, Bottino M, Cowett RM. Interventions for prevention of neonatal hyperglycemia in very low birth weight infants. *Cochrane Database Syst Rev* 2011(10):CD007615. doi: 10.1002/14651858.CD007615.pub3
4. Hays SP, Smith EO, Sunehag AL. Hyperglycemia is a risk factor for early death and morbidity in extremely low birth-weight infants. *Pediatrics* 2006;118(5):1811-8.
5. Finberg L. Dangers to infants caused by changes in osmolal concentration. *Pediatrics* 1967;40(6):1031-4.
6. Garg R, Agthe AG, Donohue PK, et al. Hyperglycemia and retinopathy of prematurity in very low birth weight infants. *J Perinatol* 2003;23(3):186-94.
7. Kao LS, Morris BH, Lally KP, et al. Hyperglycemia and morbidity and mortality in extremely low birth weight infants. *J Perinatol* 2006;Dec 26(12):730-6.
8. Yager JY. Hypoglycemic injury to the immature brain. *Clin Perinatol* 2002;29(4):651-74, vi.
9. Van den Berghe G, Wouters P, Weekers F, et al. Intensive insulin therapy in critically ill patients. *NEJM* 2001;345:1359-67.
10. Vlasselaers D, Milants I, Desmet L, et al. Intensive insulin therapy for patients in paediatric intensive care: a prospective, randomised controlled study. *Lancet* 2009;373(9663):547-56.
11. Alsweiler JM, Harding JE, Bloomfield FH. Tight glycemic control with insulin in hyperglycemic preterm babies: a randomized controlled trial. *Pediatrics* 2012;129(4):639-47.
12. Alexandrou G, Skiold B, Karlen J, et al. Early hyperglycemia is a risk factor for death and white matter reduction in preterm infants. *Pediatrics* 2010;125(3):e584-91.
13. Bottino M, Cowett RM, Sinclair JC. Interventions for treatment of neonatal hyperglycemia in very low birth weight infants. *Cochrane Database Syst Rev* 2009(1):CD007453.
14. Beardsall K. Measurement of glucose levels in the newborn. *Early Hum Dev* 2010;86(5):263-7.
15. Hirshberg E, Larsen G, Van Duker H. Alterations in glucose homeostasis in the pediatric intensive care unit: Hyperglycemia and glucose variability are associated with increased mortality and morbidity*. *Pediatr Crit Care Med* 2008
16. Latini G, De Felice C, Presta G, et al. Minimal handling and bronchopulmonary dysplasia in extremely low-birth-weight infants. *Eur J Pediatr* 2003;162(4):227-9. doi: 10.1007/s00431-002-1131-5
17. DeSalvo D, Buckingham B. Continuous glucose monitoring: current use and future directions. *Curr Diab Rep* 2013;13(5):657-62. doi: 10.1007/s11892-013-0398-4
18. Krinsley JS et al. Continuous glucose monitoring in the ICU: clinical considerations and consensus. Crit Care. 2017 Jul 31; 21(1):197. doi: 10.1186/s13054-017-1784-0.
19. Wallia A et al. Consensus Statement on Inpatient Use of Continuous Glucose Monitoring. J Diabetes Sci Technol. 2017 Apr 1:1932296817706151. doi:10.1177/1932296817706151.
20. Beardsall K, Ogilvy-Stuart AL, Ahluwalia J, et al. The continuous glucose monitoring sensor in neonatal intensive care. *Arch Dis Child Fetal Neonatal Ed* 2005;90(4):F307-10.
21. Beardsall K, Vanhaesebrouck S, Ogilvy-Stuart AL, et al. Early insulin therapy in very-low-birth-weight infants. *N Engl J Med* 2008;359(18):1873-84.
22. Uettwiller F, Chemin A, Bonnemaison E, et al. Real-time continuous glucose monitoring reduces the duration of hypoglycemia episodes: a randomized trial in very low birth weight neonates. *PLoS One* 2015;10(1):e0116255. doi: 10.1371/journal.pone.0116255

For peer review only

Appendices

Appendix 1: Paper algorithm for use in intervention arm only

Sensor Glucose mmol/l	Falling	Stable	Rising
<2.6	**Check Blood Glucose**<br>Stop any Insulin & Check all lines<br>Give additional Dextrose<br>Consider starting 20% Dextrose at 1ml/kg/hr	**Check Blood Glucose**<br>Stop any Insulin & Check all lines<br>Give additional Dextrose<br>Consider starting 20% Dextrose at 1ml/kg/hr	**Check Blood Glucose**<br>Review infusions & check lines<br>Ensure Insulin is not running<br>Consider starting/increasing<br>20% Dextrose at 1ml/kg/hr
2.6-4.0	**Check Blood Glucose**<br>Stop any Insulin & Check all lines<br>Give additional Dextrose<br>Consider starting 20% Dextrose at 1ml/kg/hr	**Check Blood Glucose**<br>Stop any Insulin & Check all lines<br>Give additional Dextrose<br>Consider starting 20% Dextrose at 1ml/kg/hr	**Observe the rate of rise**<br>Review infusions & check lines<br>Ensure Insulin is not running<br>Consider need for additional Dextrose
Target Range<br>4.0 - 8.0	**IN TARGET**<br>If the rate of fall means you will be <4.0mmol/l<br>within 1 hour consider reducing Insulin	**IN TARGET**	**IN TARGET**<br>Consider weaning any additional<br>20% Dextrose
8.0-10.0	**Observe the rate of fall**<br>Consider ***reducing*** Insulin infusion rate by 25%	Stop any additional 20% Dextrose<br>or<br>Start Insulin at 0.05 units/kg/hr<br>or<br>if Insulin is already running ***increase***<br>Insulin infusion rate by 50%	Stop any additional 20% Dextrose<br>or<br>Start Insulin at 0.05 units/kg/hr<br>or<br>if Insulin is already running ***increase***<br>Insulin infusion rate by 50%
10-15.0	**Observe the rate of fall**<br>Consider ***increasing*** Insulin infusion rate by 25%	Stop any additional 20% Dextrose<br>or<br>Start Insulin at 0.05 units/kg/hr<br>or<br>if Insulin is already running ***increase***<br>Insulin infusion rate by 50%	Stop any additional 20% Dextrose<br>or<br>Start Insulin at 0.05 units/kg/hr<br>or<br>if Insulin is already running ***increase***<br>Insulin infusion rate by 50%
> 15	**Observe the rate of fall**<br>Consider ***increasing*** Insulin infusion rate by 50%	Start Insulin at 0.05 units/kg/hr<br>**or**<br>consider ***increasing*** Insulin infusion rate<br>by 100% (that is: Double)<br>**Always check infusion lines if there is little or no response to an intervention**	Start Insulin at 0.05 units/kg/hr<br>**or**<br>consider ***increasing*** Insulin infusion rate<br>by 100% (that is: Double)<br>**Always check infusion lines if there is little or no response to an intervention**
**CRITICAL**	Please remember continuous glucose sensor readings are provided to support clinical management.<br>They provide additional information on trends in glucose levels which should be used to guide the need for blood glucose measurement.<br>Capillary/venous blood glucose levels are more accurate.<br>***Always check infusion lines if there is little or no response to an intervention***		
**CONCERN**			
**IN TARGET**			

STANDARD PROTOCOL ITEMS: RECOMMENDATIONS FOR INTERVENTIONAL TRIALS

SPIRIT 2013 Checklist: Recommended items to address in a clinical trial protocol and related documents*

Section/item	Item No	Description	Page Number on which item is reported
**Administrative information**			
Title	1	Descriptive title identifying the study design, population, interventions, and, if applicable, trial acronym	Page 1
Trial registration	2a	Trial identifier and registry name. If not yet registered, name of intended registry	Page 3
	2b	All items from the World Health Organization Trial Registration Data Set	Throughout the paper checked
Protocol version	3	Date and version identifier	Page 10
Funding	4	Sources and types of financial, material, and other support	Page 11
Roles and responsibilitie s	5a	Names, affiliations, and roles of protocol contributors	Page 11
	5b	Name and contact information for the trial sponsor	Page 11
	5c	Role of study sponsor and funders, if any, in study design; collection, management, analysis, and interpretation of data; writing of the report; and the decision to submit the report for publication, including whether they will have ultimate authority over any of these activities	Page 11
	5d	Composition, roles, and responsibilities of the coordinating centre, steering committee, endpoint adjudication committee, data management team, and other individuals or groups overseeing the trial, if applicable (see Item 21a for data monitoring committee)	Page 9 and 10
**Introduction**			

For peer review only

Background and rationale	6a	Description of research question and justification for undertaking the trial, including summary of relevant studies (published and unpublished) examining benefits and harms for each intervention	Page 4
	6b	Explanation for choice of comparators	Page 4-5
Objectives	7	Specific objectives or hypotheses	Page 4
Trial design	8	Description of trial design including type of trial (eg, parallel group, crossover, factorial, single group), allocation ratio, and framework (eg, superiority, equivalence, noninferiority, exploratory)	Page 5
**Methods: Participants, interventions, and outcomes**			
Study setting	9	Description of study settings (eg, community clinic, academic hospital) and list of countries where data will be collected. Reference to where list of study sites can be obtained	Page 5
Eligibility criteria	10	Inclusion and exclusion criteria for participants. If applicable, eligibility criteria for study centres and individuals who will perform the interventions (eg, surgeons, psychotherapists)	Page 5
Interventions	11a	Interventions for each group with sufficient detail to allow replication, including how and when they will be administered	Page 5-7
	11b	Criteria for discontinuing or modifying allocated interventions for a given trial participant (eg, drug dose change in response to harms, participant request, or improving/worsening disease)	Page 7
	11c	Strategies to improve adherence to intervention protocols, and any procedures for monitoring adherence (eg, drug tablet return, laboratory tests)	Page 9
	11d	Relevant concomitant care and interventions that are permitted or prohibited during the trial	Page 5
Outcomes	12	Primary, secondary, and other outcomes, including the specific measurement variable (eg, systolic blood pressure), analysis metric (eg, change from baseline, final value, time to event), method of aggregation (eg, median, proportion), and time point for each outcome. Explanation of the clinical relevance of chosen efficacy and harm outcomes is strongly recommended	Page 7

For peer review only - http://bmjopen.bmj.com/site/about/guidelines.xhtml

Participant timeline	13	Time schedule of enrolment, interventions (including any run-ins and washouts), assessments, and visits for participants. A schematic diagram is highly recommended (see Figure)	Page 7
Sample size	14	Estimated number of participants needed to achieve study objectives and how it was determined, including clinical and statistical assumptions supporting any sample size calculations	Page 7
Recruitment	15	Strategies for achieving adequate participant enrolment to reach target sample size	Page 7
**Methods: Assignment of interventions (for controlled trials)**			
Allocation:			
Sequence generation	16a	Method of generating the allocation sequence (eg, computer-generated random numbers), and list of any factors for stratification. To reduce predictability of a random sequence, details of any planned restriction (eg, blocking) should be provided in a separate document that is unavailable to those who enrol participants or assign interventions	Page 8
Allocation concealme nt mechanis m	16b	Mechanism of implementing the allocation sequence (eg, central telephone; sequentially numbered, opaque, sealed envelopes), describing any steps to conceal the sequence until interventions are assigned	Page 8
Implement ation	16c	Who will generate the allocation sequence, who will enrol participants, and who will assign participants to interventions	Page 8
Blinding (masking)	17a	Who will be blinded after assignment to interventions (eg, trial participants, care providers, outcome assessors, data analysts), and how	Page 7
	17b	If blinded, circumstances under which unblinding is permissible, and procedure for revealing a participant's allocated intervention during the trial	NA
**Methods: Data collection, management, and analysis**			

Data collection methods	18a	Plans for assessment and collection of outcome, baseline, and other trial data, including any related processes to promote data quality (eg, duplicate measurements, training of assessors) and a description of study instruments (eg, questionnaires, laboratory tests) along with their reliability and validity, if known. Reference to where data collection forms can be found, if not in the protocol	Page 8
	18b	Plans to promote participant retention and complete follow-up, including list of any outcome data to be collected for participants who discontinue or deviate from intervention protocols	Page 8
Data management	19	Plans for data entry, coding, security, and storage, including any related processes to promote data quality (eg, double data entry; range checks for data values). Reference to where details of data management procedures can be found, if not in the protocol	Page 8
Statistical methods	20a	Statistical methods for analysing primary and secondary outcomes. Reference to where other details of the statistical analysis plan can be found, if not in the protocol	Page 8-9
	20b	Methods for any additional analyses (eg, subgroup and adjusted analyses)	NA
	20c	Definition of analysis population relating to protocol non-adherence (eg, as randomised analysis), and any statistical methods to handle missing data (eg, multiple imputation)	Page 8
**Methods: Monitoring**			
Data monitoring	21a	Composition of data monitoring committee (DMC); summary of its role and reporting structure; statement of whether it is independent from the sponsor and competing interests; and reference to where further details about its charter can be found, if not in the protocol. Alternatively, an explanation of why a DMC is not needed	Page 9
	21b	Description of any interim analyses and stopping guidelines, including who will have access to these interim results and make the final decision to terminate the trial	Page 9

Harms	22	Plans for collecting, assessing, reporting, and managing solicited and spontaneously reported adverse events and other unintended effects of trial interventions or trial conduct	Page 9
Auditing	23	Frequency and procedures for auditing trial conduct, if any, and whether the process will be independent from investigators and the sponsor	Page 9
**Ethics and dissemination**			
Research ethics approval	24	Plans for seeking research ethics committee/institutional review board (REC/IRB) approval	Page 11
Protocol amendments	25	Plans for communicating important protocol modifications (eg, changes to eligibility criteria, outcomes, analyses) to relevant parties (eg, investigators, REC/IRBs, trial participants, trial registries, journals, regulators)	Page 10
Consent or assent	26a	Who will obtain informed consent or assent from potential trial participants or authorised surrogates, and how (see Item 32)	Page 7
	26b	Additional consent provisions for collection and use of participant data and biological specimens in ancillary studies, if applicable	NA
Confidentiality	27	How personal information about potential and enrolled participants will be collected, shared, and maintained in order to protect confidentiality before, during, and after the trial	Page 8
Declaration of interests	28	Financial and other competing interests for principal investigators for the overall trial and each study site	Page 11
Access to data	29	Statement of who will have access to the final trial dataset, and disclosure of contractual agreements that limit such access for investigators	Page 8
Ancillary and post-trial care	30	Provisions, if any, for ancillary and post-trial care, and for compensation to those who suffer harm from trial participation	NA
Dissemination policy	31a	Plans for investigators and sponsor to communicate trial results to participants, healthcare professionals, the public, and other relevant groups (eg, via publication, reporting in results databases, or other data sharing arrangements), including any publication restrictions	Page 10

For peer review only

	31b	Authorship eligibility guidelines and any intended use of professional writers	Page 10
	31c	Plans, if any, for granting public access to the full protocol, participant-level dataset, and statistical code	Page 12
**Appendices**			
Informed consent materials	32	Model consent form and other related documentation given to participants and authorised surrogates	Attached separately
Biological specimens	33	Plans for collection, laboratory evaluation, and storage of biological specimens for genetic or molecular analysis in the current trial and for future use in ancillary studies, if applicable	NA

*It is strongly recommended that this checklist be read in conjunction with the SPIRIT 2013 Explanation & Elaboration for important clarification on the items. Amendments to the protocol should be tracked and dated. The SPIRIT checklist is copyrighted by the SPIRIT Group under the Creative Commons "Attribution-NonCommercial-NoDerivs 3.0 Unported" license.

For peer review only

BMJ Open

Protocol of a Randomised Controlled Trial of Real Time Continuous Glucose Monitoring in Neonatal Intensive Care 'REACT'

Journal:	*BMJ Open*
Manuscript ID	bmjopen-2017-020816.R1
Article Type:	Protocol
Date Submitted by the Author:	12-Mar-2018
Complete List of Authors:	Beardsall, Kathryn; University of Cambridge Department of Paediatrics<br>Thomson, Lynn; University of Cambridge, Department of Paediatrics<br>Guy, Catherine; University of Cambridge, Department of Paediatrics<br>Weissenbruch, Mirjam; VU University Medical Center, P.O box 7057, 1007 MB Amsterdam, NL<br>Iglesias, Isabel; Hospital Sant Joan de Deu<br>Muthukumar, Priya; Norfolk & Norwich University Hospitals NHS Foundation Trust<br>Somisetty, Sateesh; Luton & Dunstable University Hospital<br>Bond, Simon; Cambridge University Hospitals NHS Foundation Trust, Cambridge clinical trials unit<br>Petrou, Stavros; University of Warwick, Warwick Medical School<br>Dunger, David; University of Cambridge, Paediatrics
**Primary Subject Heading**:	Paediatrics
Secondary Subject Heading:	Intensive care, Paediatrics, Evidence based practice
Keywords:	Randomised Control Trial, Glucose, Continuous monitoring, Hyperglycaemia, Hypoglycaemia

SCHOLARONE™
Manuscripts

Protocol of a Randomised Controlled Trial of Real Time Continuous Glucose Monitoring in Neonatal Intensive Care

'REACT'

Kathryn Beardsall MD FRCPCH,[1,2] Lynn Thomson RSCN,[1,2] Catherine Guy,[1] Mirjam M van Weissenbruch MD PhD,[3] Isabel Iglesias MD,[4] Priya Muthukumar MRCPCH,[5] Sateesh Kumar Somisetty MRCPCH, [6] Simon Bond,[7] Stavros Petrou[8] and David Dunger FAcad Med Sci[1]

Author Affiliations
1. University Department of Paediatrics, University of Cambridge, Box 116, Cambridge Biomedical Campus, Cambridge, CB2 0QQ, UK. 2. Cambridge University Hospitals NHS Foundation Trust, Hills Road, Cambridge, CB2 2QQ, UK. 3. VU University Medical Center, P.O box 7057, 1007 MB Amsterdam, NL 4. Sant Joan de Déu, 08950 Esplugues de Llobregat, Barcelona, Spain. 5. Norfolk & Norwich University Hospitals NHS Foundation Trust, Colney Lane, Norwich, NR4 7UY, UK. 6. Luton & Dunstable University Hospital, Lewsey Road, Luton LU4 0DZ, UK. 7. Cambridge Clinical Trials Unit, Cambridge University Hospitals NHS Foundation Trust, Cambridge, CB2 0QQ, UK. 8. Division of Health Sciences, Warwick Medical School, University of Warwick, Coventry, CV4 7AL, UK

Addresss for Correspondence:
Dr K Beardsall email: kb274@cam.ac.uk 01223 746791

Short Title: RCT of Real time CGM in NICU

Key words:
randomised controlled trial, glucose, continuous monitoring, hyperglycaemia, hypoglycaemia

Abbreviations
BG blood glucose, CGM continuous glucose monitoring, DMC Data monitoring committee, NICU neonatal intensive care unit, SG Sensor glucose, TSC Trial Steering committee

For peer review only

The Corresponding Author has the right to grant on behalf of all authors and does grant on behalf of all authors, a worldwide licence to the Publishers and its licensees in perpetuity, in all forms, formats and media (whether known now or created in the future), to i) publish, reproduce, distribute, display and store the Contribution, ii) translate the Contribution into other languages, create adaptations, reprints, include within collections and create summaries, extracts and/or, abstracts of the Contribution, iii) create any other derivative work(s) based on the Contribution, iv) to exploit all subsidiary rights in the Contribution, v) the inclusion of electronic links from the Contribution to third party material where-ever it may be located; and, vi) licence any third party to do any or all of the above."

"All authors have completed the ICMJE uniform disclosure form at www.icmje.org/coi_disclosure.pdf and declare: KB on behalf of the research team has received NIHR EME grant and provision of equipment from Medtronic and Nova Biomed to support this trial. There are no other relationships or activities that could appear to have influenced the submitted work."

For peer review only

Abstract

Introduction: Hyperglycaemia is common in the very preterm infant and has been associated with adverse outcomes. Preventing hyperglycaemia without increasing the risk of hypoglycaemia has proved challenging. The development of real time continuous glucose monitors (CGM) to inform treatment decisions provides an opportunity to reduce this risk. This study aims to assess the feasibility of CGM combined with a specifically designed paper guideline to target glucose control in the preterm infant.

Methods and Analyses: The REACT Randomised controlled Trial (Real Time Continuous Glucose Monitoring in Neonatal Intensive Care) is an international multicentre randomised controlled trial. Two hundred preterm infants ≤1200g, and ≤24 hours of age will be randomly allocated to either real time continuous glucose monitoring, or standard care (with blinded CGM data collection). The primary outcome is time in target 2.6-10mmol/l during the study intervention assessed using continuous glucose monitoring. Secondary outcomes include efficacy relating to glucose control, utility including staff acceptability, safety outcomes relating to incidence and prevalence of hypoglycaemia and health economic analyses.

Ethics and Dissemination: The REACT Trial has been approved by the NHS Health Research Authority National Research Ethics Service Committee East of England (Cambridge Central); Medical Ethics Review Committee, VU University Medical Centre, Amsterdam, The Netherlands and the Research Ethics Committee, Sant Joan de Déu Research Foundation, Barcelona, Spain. Recruitment began in July 2016 and will continue until mid 2018. The Trial has been adopted by the National Institute of Health Research Clinical Research Network portfolio (ID: 18826) and is registered with the ISRCTN registry (ID: 12793535). Dissemination plans include presentations at scientific conferences, scientific publications and efforts at stakeholder engagement.

For peer review only

Strengths and Limitations

The comparison of real time CGM data with blinded CGM data in the control study arm will provide detailed comparable data on efficacy and safety between study arms.

As an International multicentre trial the results will be generalizable across a range of neonatal intensive care settings.

Input by staff and parents within the trial itself as well as part of the Trial management will provide information on utility and facilitate translation of the outcomes into clinical practice.

The study requires recruitment within 24 hours of preterm birth, which requires a significant commitment from the clinical and research teams if it is to be successful.

The study is powered to detect a difference in the Primary outcome 'Time in target' (2.6-10mmol/l), but will not have the power to detect the impact on clinical outcomes.

For peer review only

Introduction

In utero, glucose levels are normally maintained between 4-6mmol/l [1], but infants born preterm are at risk of both hyperglycaemia and hypoglycaemia[2]. Hyperglycaemia and hypoglycaemia have both been associated with increased mortality and morbidity of preterm babies[3,4]. Hyperglycaemia can lead to acute problems of a persistent osmotic diuresis and metabolic acidosis which can be difficult to control and has been associated with increased risk of intraventricular haemorrhage and patent ductus arteriousus[5]. Hyperglycaemia has also been associated with increased long term morbidity, including increased risk of retinopathy of prematurity[4 6-8]. Hypoglycaemia is associated with characteristic occipital temporal lesions[8]. Attempts to reduce risks associated with hyperglycaemia in adult and paediatric and neonatal intensive care (NICU) have resulted in increased risk of hypoglycaemia[9 10 11]. This is of particular concern for the very preterm infant in whom there is very varied insulin sensitivity, which increases the risk of hypoglycaemia. In addition the developing brain appears to be particularly vulnerable to both hyperglycaemic [12], and hypoglycaemic insults. Early postnatal glucose control may be an important modifiable risk factor for clinical outcomes. A recent Cochrane review has highlighted the need for further studies into the impact of interventions to improve glucose control in these infants[13].

Managing glucose control is dependent in part on methods of measuring and monitoring glucose levels. Within NICU glucose measurements are currently limited to intermittent blood sampling [14], with long periods when glucose levels are unknown. In contrast, other physiological parameters such as oxygen saturation, blood pressure and heart rate are all monitored continuously to prevent wide fluctuations. It is increasingly thought that fluctuations in glucose levels may also have a significant impact on long term outcomes [15]. The reason for the intermittent measurement is that current methodologies rely on blood sampling either from a central arterial line or by heel prick. Clinical care for preterm infants aims to reduce the frequency of handling [16] and volume of blood sampled as this has been shown to improve outcomes.

Developments in the measurement of glucose levels in patients with diabetes mellitus include continuous glucose monitoring of interstitial glucose levels [17]. Real time data on interstitial glucose levels now provides information on glucose trends with the potential for earlier intervention and prevention of both hyperglycaemia and hypoglycaemia. Benefits within adult intensive care remain controversial [18], however the benefits in the setting of NICU may be more marked as blood glucose (BG) measurements are taken much less frequently in these small babies. There have also been key developments in the technology including extended life of sensors (previously 72 hours) and improved accuracy [19]. The latter is particularly relevant due to the threshold levels of hypoglycaemia and hyperglycaemia (<2.6 and >10mmol/L) which are more extreme than in adults and are at the limits of accuracy of many methods of glucose measurement.

Blinded CGM has been used in preterm babies within clinical trials[2 20 21], and studies of real time devices have shown a benefit in providing early warning of and prevention of hypoglycaemia [22]. Studies have not so far attempted to use CGM to guide clinical management to support the targeting of glucose control in preterm infants. A single centre feasibility study of the real time monitors demonstrated that sensor glucose (SG) values are comparable with BG values (REACT feasibility study REC Ref: 14/EE/0127). Data from the feasibility has helped to design this randomised controlled trial. This multicentre randomised controlled trial will determine whether real time continuous glucose monitoring, with support from a paper algorithm, can help improve the management of glucose control in terms of efficacy, safety and clinical acceptability. This will not only enhance the short term management of glucose control in infants requiring intensive care but by reducing the risks associated with both hyperglycaemia and hypoglycaemia may impact on long term clinical outcomes.

Trial Objectives

The REACT Trial will evaluate efficacy, safety, utility and cost effectiveness of real time continuous glucose monitoring (CGM) in preterm infants in NICU. Our primary hypothesis is that the use of real time CGM will improve the time a baby's glucose levels (measured using CGM)

For peer review only - http://bmjopen.bmj.com/site/about/guidelines.xhtml

remain within the target 2.6-10mmol/l (widely accepted clinical target for glucose control), compared to standard clinical practice (with blinded CGM data collection). Secondary objectives include evaluation of clinical acceptability in the preterm infant (using staff and parent questionnaires) and safety in relation to the device itself and risk of hypoglycaemia.

Methods

Study Design

This is a multicentre interventional, randomised controlled trial of CGM compared to standard clinical management (control). We will recruit potential participants within 24 hours of birth and continually monitor their glucose levels for 6 days. Data will be collected until 36 weeks corrected gestational age. No other aspects of concomitant care are prohibited during the trial.

Eligibility Criteria

Inclusion Criteria

Infants who have a birth weight ≤ 1200g, are ≤ 24 hours of age, ≤ 33+6 weeks gestation and in whom written informed parental consent has been received.

Exclusion Criteria

Any lethal congenital abnormality known at trial entry, any congenital metabolic disorder known at trial entry and any neonates who, in the opinion of the treating clinician at trial entry, have no realistic prospect of survival.

Intervention

Babies will be randomised in a 1:1 ratio, into control and intervention arms of the study using a web randomisation system, TENALEA. This is an open study in which the clinical staff, research team and parents will be aware of the study arm and intervention. All babies will have a subcutaneous sensor inserted, Enlite™(Medtronic) that will be linked to a Medtronic MiniMed 640G™ System and will be calibrated with point of care BG levels. For consistency across sites calibration will be standardized by providing all units with Nova StatStrip™ meters.

Standard care with blinded CGM data collection (Control)

These infants will have their glucose control monitored and managed according to standard clinical practice using intermittently sampled BG levels. Nutritional delivery (including glucose) and insulin delivery will be prescribed according to the standard clinical guidelines within each unit. The CGM device will collect glucose data continuously but the clinical team will be blinded to the data as the monitor will be kept covered and fastened with a tamper proof seal.

Real Time Continuous Glucose Monitoring Device with paper algorithm (Intervention)

The CGM data will be open to view by the clinical team during the first week of the baby's life and the staff will be advised to read and record the SG data hourly as part of standard clinical monitoring. This will support staff to use the additional data available from real time monitoring to guide timing of BG measurement and changes in clinical management. Interventions to target glucose control will then be guided by the specifically designed paper algorithm. This algorithm was developed during the REACT feasibility study (REC Ref: 14/EE/0127).

Medical Devices: MiniMed 640G System

The MiniMed 640G System is indicated for glucose monitoring and for continuous delivery of insulin, for the management of diabetes mellitus in persons requiring insulin. Monitoring equipment only will be used for this study. The system being used comprises linking the Enlite sensor

For peer review only

(Medtronic, Northridge, CA, USA) using the Guardian 2 Link transmitter to the MiniMed 640G which then displays the glucose data in real time.

i. Sensor

The Enlite sensor (Medtronic, Northridge, CA, USA) is a CGM sensor which received CE mark in 2013 (CE certificate No. 21024). The sensor (figure 1) comprises a disposable subcutaneous oxidase-based platinum electrode that catalyses interstitial glucose generating an electrical current every 10 seconds which is transmitted to a monitor for display and/or recording. The data will be recorded and/or displayed as an averaged value every 5 minutes, giving a total of 288 readings per day. Glucose values outside the range 2.2-24.0 mmol/l (40-430 mg/dl) are reported as < 2.2 mmol/l (40 mg/dl), or >24 mmol/l (430 mg/dl) respectively.

The sensor will be inserted subcutaneously (into the thigh) by hand, not using the standard insertion device, thus ensuring the sensor is inserted into the subcutaneous tissue. The sensors are soft and flexible, approximately 8.75mm in length and are mounted inside a hollow needle to allow for subcutaneous insertion. Once the sensor is inserted the introducer needle will be withdrawn, and the sensor attached to a small Guardian™ 2 Link transmitter (CE Mark 2013; Certificate No. 8858) for data transfer to the MiniMed 640G System for data viewing. The sensor will then be secured with a clear occlusive dressing (again trimmed to ensure minimal contact with the infant's skin), so that the insertion site can be inspected daily. A blood sample will be required every 12 hours to ensure calibration of the sensor. Sensors will be removed after 7 days.

ii. ***Guardian 2 Link Transmitter*** (Figure 2)

It connects to the glucose sensor and sends glucose data wirelessly to the MiniMed® 640G monitor (pump).

iii. ***The MiniMed 640G/monitor (pump)*** (Figure 3)

The 640G monitor as well as providing continuous glucose values will store data so that it can be analysed to track patterns and improve glucose management. Glucose data will be downloaded from the 640G at the end of the study period to a computer for analyses. This device received CE mark in 2014 (CE certificate No. 8857).

Blinding

To ensure that the real time data is not available to staff caring for babies in the control arm of the study the CGM will be kept secure in an opaque bag with tamper tag seals. The bags will be opened for calibration every 12 hours, and each baby will have a log kept of timings of when the tamper tag is broken/resealed.

Outcomes

The *Primary outcome* is percentage of time SG in target range of 2.6-10mmol/l. This was selected after consultation as it represents internationally, the clinically most widely accepted target range for glucose control for this population.
Secondary Outcomes have been selected to provide further evidence around efficacy, acceptability and safety. These include for efficacy:

- mean SG
- percentage of time SG in range of 4-8mmol/l
- SG variability within individuals as assessed by within patient standard deviation
- percentage of time glucose levels in hyperglycaemic range, SG >15mmol/l.

For peer review only

Acceptability will be assessed by a specifically designed staff questionnaire which will be completed anonymously on day 3 and 7 by the clinical team caring for the baby as well as a parent questionnaire on day 7.

Safety outcomes include both measures of BG and SG to address potential differences in methodologies and to provide data on prevalence of exposures that may be undetected clinically in the control arm of the study:

- Incidence of hypoglycaemia defined as any episode of BG >2.2mmol/l and <2.6mmol/l
- Incidence of hypoglycaemia defined as continuous episode of CGM SG <2.6mmol/l for >1hour
- Incidence of severe hypoglycaemia defined as any episode of BG ≤2.2mmol/l

Health Economics: cost-effectiveness will be expressed in terms of incremental cost per additional case of adequate glucose control between 2.6mmol/l – 10mmol/l

Sample Size

Based on data from the REACT feasibility study and historical control data, we conservatively assume that the SD of the primary endpoint is 22%.   A sample size of 200 participants will enable a treatment effect of a 10% increase in the mean value of the primary endpoint to be detected with 90% power using a two-sided 5% significance test in the primary analysis. Based on a consensus of expert opinion a difference of 10% is believed to be of minimal clinical relevance. It is expected that a small number of patients will be withdrawn from the study. Reasons for these withdrawals include transfer to participant’s local NICU, withdrawal of parental consent or death.

Recruitment Plan

All babies will be recruited within 24 hours of birth at one of the NICUs that has been approved for study participation. Due to the short time frame from birth to recruitment potentially eligible babies will be identified in a number of ways: i. liaison with the obstetric team to highlight mothers at risk of preterm delivery, ii. Liaison with the neonatal transport team to identify babies who have been born in local units being transferred to a study centre, iii. Liaison with the NICU clinical team of study centres. Screening of eligible patients will be undertaken in collaboration with the clinical team and families approached only if considered eligible and the families consent to being approached about study involvement. Screening logs will be reviewed regularly by the coordinating centre to identify any issues around recruitment.

The REACT recruitment centres are level 3 NICUs. They have been selected either because of their previous experience of using continuous glucose monitoring in the preterm infant, or they are centres with a proportionately large number of babies which would fulfil the study inclusion criteria and represent an International range of clinical practice and thus provide generalisability of the intervention. The first patient was recruited in July 2016, and the end of study is planned for November 2018.

Randomisation

Randomisation will take place within 24 hours of delivery and babies will be randomised in a 1:1 ratio into control and intervention arms of the study using a central web randomisation system, TENALEA. The randomisation will use blocked stratified randomisation. The stratification factors will be to recruiting centres and gestation (<26 weeks gestation, ≥26 weeks gestation). The programme will notify the local research team of treatment allocation who will then inform their clinical team regarding the practicalities of management. This is an open study in which the clinical and research teams and parents will be aware of the study arm and intervention.

Data management and analyses

Data collection will be undertaken from birth to 36 weeks corrected gestational age. If a baby has been discharged from their recruiting NICU the research team will use local and national

For peer review only

databases, local contacts and links with parents to ensure complete follow up data is obtained. All data will be sent to the coordinating centre in Cambridge where it will be entered onto a MACRO database. All data will be collected, transferred and stored to comply with GCP and Data Protection legislation. Access to data will only be granted to authorised personnel involved in study management or for auditing/monitoring to comply with regulations. To maintain high quality standard of data entry the data base will be tested and validated prior to use.

Efficacy: This will be assessed by comparison of data collected by real time CGM in the intervention arm and blinded CGM in the control infants.
Clinical Acceptability: Parents, nurses and medical staff, caring for babies in the study will be asked to complete study specific questionnaires.
Safety: This will be assessed in 3 areas: incidence of hypoglycaemia measured as part of clinical care (BG levels) and after review of SG data; device safety through adverse device effect reporting; and acute mortality and morbidity outcomes as part of the CRF.
Costs for Economic Evaluation: Data will be collected on the health service resources used in the treatment of infants during the period between randomisation and 36 weeks gestation and based on British Association of Perinatal Medicine standard criteria for level of care, as well as neonatal complications. Current UK unit costs will be applied to each resource item and a *per diem* cost for each level of neonatal care will be based on Department of Health reference costs calculated on a full absorption costing basis.

Data Analyses

The primary endpoint will be analysed using linear regression to estimate the absolute difference in time SG in target of 2.6 - 10mmol/l, adjusting for baseline variables (centre, gestation). Analyses will be undertaken both for intention to treat and as treated populations. Estimates of treatment effect, with 95% confidence intervals and p-values will be provided. Secondary endpoints that are continuous variables will be analysed in a similar fashion. Secondary endpoints that are counts or binary variables will be analysed using an appropriate regression framework. Methods will be used to reduce the likelihood of a type I error. All of the efficacy endpoints will be ranked in order of importance: mean SG, percentage of time SG in target of 4-8mmol/l, SG variability within individuals as assessed by within-patient standard deviation, percentage of time glucose levels in hyperglycaemic range - SG >15mmol/l.

Continuous variables will report the mean, median, SD, range, max and min. Binary or categorical endpoints will be represented using frequency tables in the “p% (r/n)” format. The analysis will look for a treatment interaction effect with the following baseline variables: centre, sex, corrected gestational age, birth weight SDS, use of antenatal steroids, maternal chorioamnionitis and maternal diabetes using the regression framework in an exploratory, non-confirmatory manner.

An incremental cost-effectiveness analysis will be performed. In the baseline analysis, the economic evaluation will be expressed as the incremental cost per additional case of adequate glucose control. Adequate control will be considered as 80% of time in target. Given the multinational nature of the trial, the hierarchical structures of the cost and outcomes data will be taken into account in the analysis plan. Due to the known limitations of within-trial economic evaluations we will also construct a decision-analytical model to model the cost-effectiveness of CGM beyond the time horizon of the trial.

Site Training

Research teams at each site will be required to have up to date GCP training and have undertaken training in study procedures including use of the CGM and Nova Biomedical point of care devices. Paper and online resources as well as a call line will be available to support the research teams.

Monitoring

For peer review only - http://bmjopen.bmj.com/site/about/guidelines.xhtml

Data returns will be continually monitored by the central team for completeness and timeliness of all data returned. Compliance with intervention strategy in each study arm will also be reviewed to ensure there is not ‘crossover’ between study arms. A monitoring plan is in place determining frequency and scope of site monitoring based on continuing risk review. Face-to-face monitoring visits will initially be undertaken within the first 6 months and then adjusted following assessment of recruitment rate, number of data queries and SAE reports. The study sites will provide direct access to all trial related source data and reports for the purpose of monitoring and auditing by the central study team, Sponsor and regulatory authorities as required.

Data and safety monitoring

The Data and Safety Monitoring Committee (DMC) is responsible for safeguarding the interests of the trial participants and making recommendations to the Trial Steering Committee (TSC). The REACT DMC roles and responsibilities and operating procedures are defined in the REACT DMC Charter. It is composed of a 3 independent multidisciplinary experts who are not involved in the conduct of the trial in any way. They met prior to the initiation of enrolment and determined a plan to review the protocol, compliance, safety and adverse events and outcome data after a pre-specified number of babies have been recruited. The TSC is comprised of 5-6 independent members and has a Charter defining the member’s roles and responsibilities. The TSC provide advice, through its Chair, to the Chief Investigator, and report to Trial Sponsor and Trial Funder.

Safety will be assessed continuously during each baby’s stay in NICU. The frequency of Adverse Event (AE) and Serious Adverse Event (SAE) as defined by The International Conference on Harmonisation and that would normally require reporting within a clinical trial is anticipated to be high in the population being studied despite the low risk of the study intervention. Following discussions with the Medicines and Healthcare Products Regulatory Agency (MHRA) and in accordance with regulatory guidance which allows for exceptions in such circumstances a modified reporting plan was agreed.

Any Adverse Device Effect (ADE) will be recorded and reported to the coordinating centre. All device deficiencies that might have led to a Serious Adverse Device Effect (SADE) if suitable action had not been taken; intervention had not been made; or if circumstances had been less fortunate, will be reported to the Sponsor as for SAEs/SADEs. Adverse events will be recorded in the notes and some will be captured as exploratory outcomes as part of the CRF. SAEs are common in this population, therefore the MHRA requested the following expectation for safety reporting:

During the Intervention period of the study (study days 1-7)

The following expected SAEs will need to be recorded in the CRF (safety log) and reported using the safety report form to the sponsor within 24 hours of awareness of the event: i. Death, ii. Culture positive infection, iii. Severe hypoglycaemia (<2.6mmol/l), iv. Seizures, v. any other related SAE.

Post intervention period (study day 7 until end of study)

Important medical outcomes for the trial will be captured in the CRF at the 36 weeks corrected gestation assessment. Other SAEs are anticipated events for this study population and do not need to be recorded or reported separately as an SAE if judged by the clinical team to be unrelated to the study.

Ethics and Dissemination

The investigator or a suitably qualified person designated by the principal investigator will receive written informed consent from the patient’s parent/legally acceptable representative before any trial-specific activity is performed. The Trial Protocol (version 4, 7 Nov 2016) has been approved by the NHS Health Research Authority National Research Ethics Service Committee East of England – Cambridge Central (REC REF 15/EE/0158). Clinical Trial authorisation has been granted by the MHRA (REF: CI/2016/0011). Written approvals will be received from individual hospital sites prior to recruitment. Approvals have also been obtained from Medical Ethics Review

For peer review only - http://bmjopen.bmj.com/site/about/guidelines.xhtml

Committee, VU University Medical Centre, Amsterdam and Heath Care Inspectorate (REF: 2017-1 398434/VIO 14949), The Netherlands and the Research Ethics Committee, Sant Joan de Déu Research Foundation, Barcelona, and Ministry of Health Social Services and Equality (REF: 591/16/EC), Spain. The Chief Investigator will ensure that the trial is conducted in accordance with the principles of the Declaration of Helsinki and in conformity with the Medical Devices Regulations and any relevant amendments. The findings of the Trial will be prepared and presented at national and International meetings and conferences and published in peer reviewed journals by the academic team

Patient and Public Involvement

Consultation with the parents from the local parent support group was undertaken to help inform trial design. Parents are being asked to provide feedback on study involvement as part of the protocol. The TSC includes a lay person. We will send newsletters to parents to update them on study progress.

Conclusions

The REACT trial is an international multicentre trial which will randomise 200 preterm babies (≤1200g and ≤24 hours of age) to receive either real time continuous glucose monitoring or standard clinical management of glucose control (with blinded CGM data collection). This study will determine if real time CGM can support better targeting of glucose control in these babies reducing the risk of both hyperglycaemia and hypoglycaemia. This has the potential to impact on both the acute management, but also in the future on outcomes of preterm babies who are at risk from glucose dysregulation.

For peer review only

Acknowledgements

This project is funded by the Efficacy and Mechanism Evaluation (EME) Programme, an MRC and NIHR partnership and supported in terms of statistical support and data management by the Cambridge Clinical Trials Unit.

The authors are thankful for the support of the Cambridge NICU team who helped to shape the RCT design based on feedback from the feasibility study and members of the Department of Paediatrics in Cambridge for support with trial set up. They also wish to thank all the families that are currently and will be taking part in the study. It is also important to acknowledge all the members of the clinical teams who help to identify potentially eligible babies but moreover take the time and effort within a busy clinical environment to take on the additional engagement with the practicalities of a new study and new way of caring for babies. We also need to acknowledge the independent members of the TSC and DMEC who helped to finalise the protocol. REACT is a portfolio adopted study and receives support from the UK Children Research Network.

Contributors

The trial was conceptualised by KB and DBD with input from LT. II, PM, SS, SP, MW, SB are named Investigators on the REACT Trial. All authors contributed to design of the trial with specific additional contributions from each co-author within their area of expertise. KB is the lead doctor for the trial and prepared the first version and subsequent revisions of the manuscript. LT is the lead nurse for the trial and CG trial coordinator. All authors contributed to the manuscript and have approved the final manuscript prior to submission.

Collaborators/Study Investigators

REACT Investigators: Professor David Dunger, Dr Roman Hovorka, Dr Sateesh Somisetty, Dr Priya Muthukumar, Dr Mirjiam Weissenbruch, Dr Amanda Ogilvy Stuart and Dr Isabel Iglesias Platas.

Funding

The study is funded by NIHR: Evaluation, Trials and Studies Programme. Further support was provided by Medtronic for supplying MiniMed 640G System, Enlite sensors and Guardian 2 Link transmitters and Nova Biomedical provided all point of care equipment and training for glucose, lactate and ketone measurement. The trial is jointly sponsored by Cambridge University Hospitals NHS Foundation Trust and University of Cambridge.

Disclaimer

The Trial Sponsor and funders (NIHR and Medtronic) had no role in trial design, and will have no role in gathering of data, access to data, preparation of the manuscript or decision to publish the results. The views expressed in this publication are those of the author(s) and not necessarily those of the MRC, NHS, NIHR or the Department of Health.

The EME Programme is funded by the MRC and NIHR, with contributions from the CSO in Scotland and NISCHR in Wales and the HSC R&D Division, Public Health Agency in Northern Ireland.

Competing Interests

Dr Bond reports grants from NIHR, during the conduct of the study.

Ethics Approval

The Trial has been approved by the NHS Health Research Authority National Research Ethics Service Committee East of England – Cambridge Central (REC REF 15/EE/0158).

Provenance and Peer review

Not commissioned; externally peer reviewed

Open Access

For peer review only

This is an open access article distributed in accordance with the terms of the Creative Commons Attribution (CC BY 4.0) license, which permits others to distribute, remix adapt and build upon this work, for commercial use, provided the original work is properly cited.

For peer review only

References

1. Economides DL, Nicolaides KH, Campbell S. Metabolic and endocrine findings in appropriate and small for gestational age fetuses. *J Perinat Med* 1991;19(1-2):97-105.
2. Beardsall K, Vanhaesebrouck S, Ogilvy-Stuart AL, et al. Prevalence and determinants of hyperglycemia in very low birth weight infants: cohort analyses of the NIRTURE study. *J Pediatr* 2010;157(5):715-9 e1-3. doi: 10.1016/j.jpeds.2010.04.032
3. Sinclair JC, Bottino M, Cowett RM. Interventions for prevention of neonatal hyperglycemia in very low birth weight infants. *Cochrane Database Syst Rev* 2011(10):CD007615. doi: 10.1002/14651858.CD007615.pub3
4. Hays SP, Smith EO, Sunehag AL. Hyperglycemia is a risk factor for early death and morbidity in extremely low birth-weight infants. *Pediatrics* 2006;118(5):1811-8.
5. Finberg L. Dangers to infants caused by changes in osmolal concentration. *Pediatrics* 1967;40(6):1031-4.
6. Garg R, Agthe AG, Donohue PK, et al. Hyperglycemia and retinopathy of prematurity in very low birth weight infants. *J Perinatol* 2003;23(3):186-94.
7. Kao LS, Morris BH, Lally KP, et al. Hyperglycemia and morbidity and mortality in extremely low birth weight infants. *J Perinatol* 2006;Dec 26(12):730-6.
8. Yager JY. Hypoglycemic injury to the immature brain. *Clin Perinatol* 2002;29(4):651-74, vi.
9. Van den Berghe G, Wouters P, Weekers F, et al. Intensive insulin therapy in critically ill patients. *NEJM* 2001;345:1359-67.
10. Vlasselaers D, Milants I, Desmet L, et al. Intensive insulin therapy for patients in paediatric intensive care: a prospective, randomised controlled study. *Lancet* 2009;373(9663):547-56.
11. Alsweiler JM, Harding JE, Bloomfield FH. Tight glycemic control with insulin in hyperglycemic preterm babies: a randomized controlled trial. *Pediatrics* 2012;129(4):639-47.
12. Alexandrou G, Skiold B, Karlen J, et al. Early hyperglycemia is a risk factor for death and white matter reduction in preterm infants. *Pediatrics* 2010;125(3):e584-91.
13. Bottino M, Cowett RM, Sinclair JC. Interventions for treatment of neonatal hyperglycemia in very low birth weight infants. *Cochrane Database Syst Rev* 2009(1):CD007453.
14. Beardsall K. Measurement of glucose levels in the newborn. *Early Hum Dev* 2010;86(5):263-7.
15. Hirshberg E, Larsen G, Van Duker H. Alterations in glucose homeostasis in the pediatric intensive care unit: Hyperglycemia and glucose variability are associated with increased mortality and morbidity*. *Pediatr Crit Care Med* 2008
16. Latini G, De Felice C, Presta G, et al. Minimal handling and bronchopulmonary dysplasia in extremely low-birth-weight infants. *Eur J Pediatr* 2003;162(4):227-9. doi: 10.1007/s00431-002-1131-5
17. DeSalvo D, Buckingham B. Continuous glucose monitoring: current use and future directions. *Curr Diab Rep* 2013;13(5):657-62. doi: 10.1007/s11892-013-0398-4
18. Krinsley JS et al. Continuous glucose monitoring in the ICU: clinical considerations and consensus. Crit Care. 2017 Jul 31; 21(1):197. doi: 10.1186/s13054-017-1784-0.
19. Wallia A et al. Consensus Statement on Inpatient Use of Continuous Glucose Monitoring. J Diabetes Sci Technol. 2017 Apr 1:1932296817706151. doi:10.1177/1932296817706151.
20. Beardsall K, Ogilvy-Stuart AL, Ahluwalia J, et al. The continuous glucose monitoring sensor in neonatal intensive care. *Arch Dis Child Fetal Neonatal Ed* 2005;90(4):F307-10.
21. Beardsall K, Vanhaesebrouck S, Ogilvy-Stuart AL, et al. Early insulin therapy in very-low-birth-weight infants. *N Engl J Med* 2008;359(18):1873-84.
22. Uettwiller F, Chemin A, Bonnemaison E, et al. Real-time continuous glucose monitoring reduces the duration of hypoglycemia episodes: a randomized trial in very low birth weight neonates. *PLoS One* 2015;10(1):e0116255. doi: 10.1371/journal.pone.0116255

Figure Legends

Figure 1: The Enlite™ glucose sensor (Medtronic, Watford UK)

Figure 2: The Enlite™ glucose sensor with The Guardian™ 2 Link transmitter attached

For peer review only - http://bmjopen.bmj.com/site/about/guidelines.xhtml

Figure 3: MiniMed™ 640G (Medtronic, Watford UK)

For peer review only

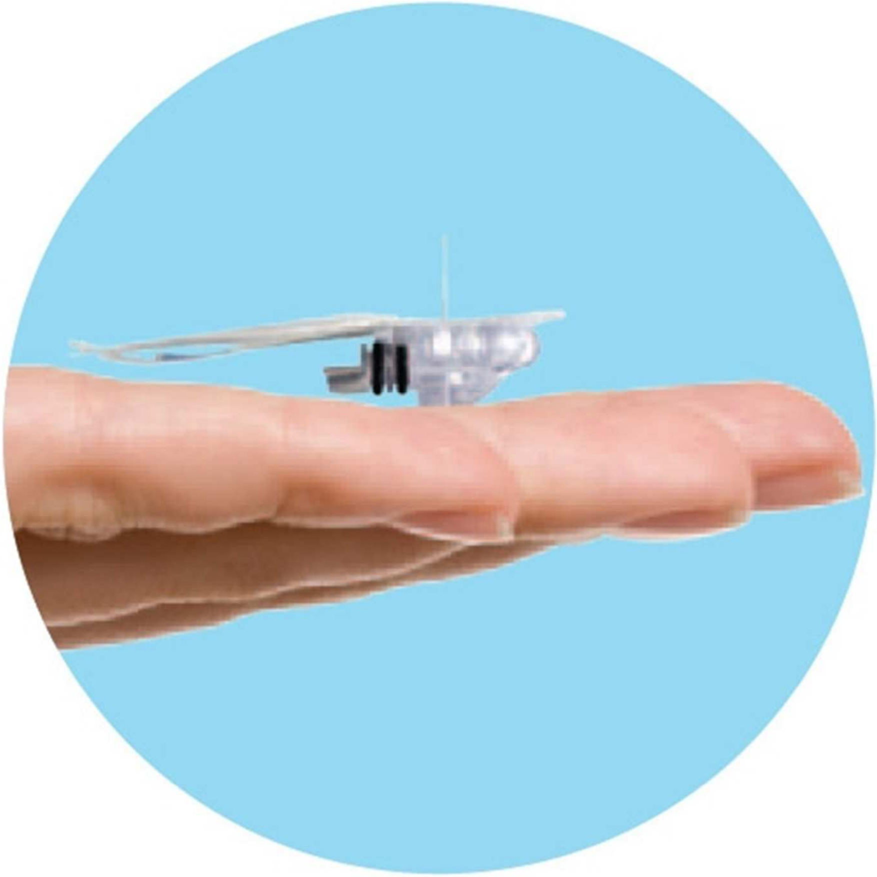

Figure 1: The Enlite™ glucose sensor (Medtronic, Watford UK)

48x42mm (300 x 300 DPI)

For peer review only - http://bmjopen.bmj.com/site/about/guidelines.xhtml

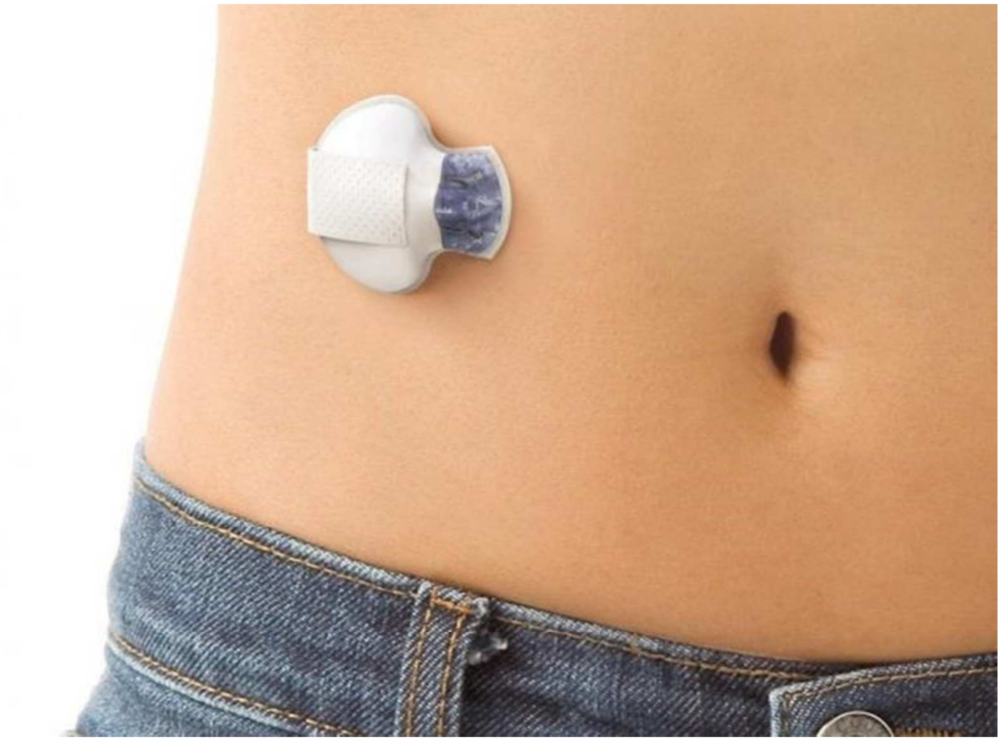

Figure 2: The Enlite™ glucose sensor with The Guardian™ 2 Link transmitter attached

168x123mm (96 x 96 DPI)

For peer review only

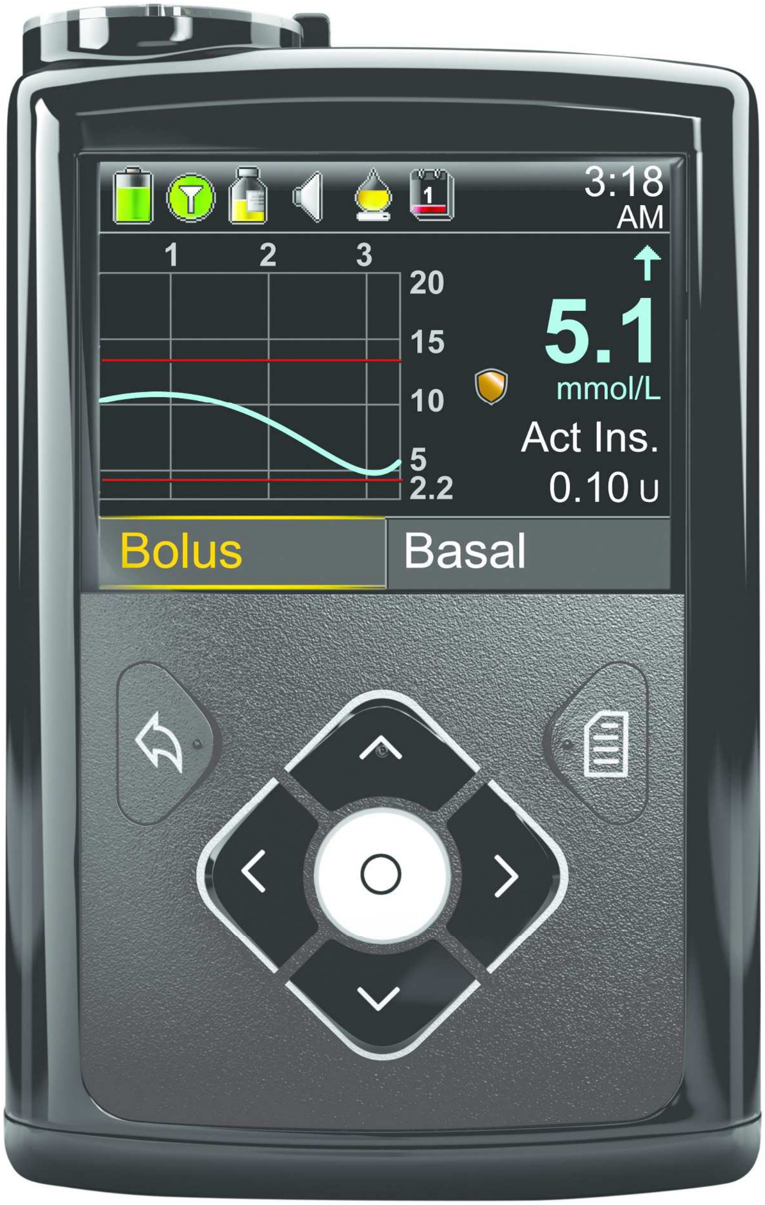

Figure 3: MiniMed™ 640G (Medtronic, Watford UK)

150x237mm (300 x 300 DPI)

Appendices

Appendix 1: Paper algorithm for use in intervention arm only

Sensor Glucose mmol/l	Falling	Stable	Rising
<2.6	**Check Blood Glucose**<br>Stop any Insulin & Check all lines<br>Give additional Dextrose<br>Consider starting 20% Dextrose at 1ml/kg/hr	**Check Blood Glucose**<br>Stop any Insulin & Check all lines<br>Give additional Dextrose<br>Consider starting 20% Dextrose at 1ml/kg/hr	**Check Blood Glucose**<br>Review infusions & check lines<br>Ensure Insulin is not running<br>Consider starting/increasing<br>20% Dextrose at 1ml/kg/hr
2.6-4.0	**Check Blood Glucose**<br>Stop any Insulin & Check all lines<br>Give additional Dextrose<br>Consider starting 20% Dextrose at 1ml/kg/hr	**Check Blood Glucose**<br>Stop any Insulin & Check all lines<br>Give additional Dextrose<br>Consider starting 20% Dextrose at 1ml/kg/hr	**Observe the rate of rise**<br>Review infusions & check lines<br>Ensure Insulin is not running<br>Consider need for additional Dextrose
Target Range<br>4.0 - 8.0	**IN TARGET**<br>If the rate of fall means you will be <4.0mmol/l within 1 hour consider reducing Insulin	**IN TARGET**	**IN TARGET**<br>Consider weaning any additional 20% Dextrose
8.0-10.0	**Observe the rate of fall**<br>Consider ***reducing*** Insulin infusion rate by 25%	Stop any additional 20% Dextrose<br>or<br>Start Insulin at 0.05 units/kg/hr<br>or<br>if Insulin is already running ***increase***<br>Insulin infusion rate by 50%	Stop any additional 20% Dextrose<br>or<br>Start Insulin at 0.05 units/kg/hr<br>or<br>if Insulin is already running ***increase***<br>Insulin infusion rate by 50%
10-15.0	**Observe the rate of fall**<br>Consider ***increasing*** Insulin infusion rate by 25%	Stop any additional 20% Dextrose<br>or<br>Start Insulin at 0.05 units/kg/hr<br>or<br>if Insulin is already running ***increase***<br>Insulin infusion rate by 50%	Stop any additional 20% Dextrose<br>or<br>Start Insulin at 0.05 units/kg/hr<br>or<br>if Insulin is already running ***increase***<br>Insulin infusion rate by 50%
> 15	**Observe the rate of fall**<br>Consider ***increasing*** Insulin infusion rate by 50%	Start Insulin at 0.05 units/kg/hr<br>**or**<br>consider ***increasing*** Insulin infusion rate<br>by 100% (that is: Double)<br>**Always check infusion lines if there is little or no response to an intervention**	Start Insulin at 0.05 units/kg/hr<br>**or**<br>consider ***increasing*** Insulin infusion rate<br>by 100% (that is: Double)<br>**Always check infusion lines if there is little or no response to an intervention**
**CRITICAL**	Please remember continuous glucose sensor readings are provided to support clinical management.<br>They provide additional information on trends in glucose levels which should be used to guide the need for blood glucose measurement.<br>Capillary/venous blood glucose levels are more accurate.<br>***Always check infusion lines if there is little or no response to an intervention***		
**CONCERN**			
**IN TARGET**			

STANDARD PROTOCOL ITEMS: RECOMMENDATIONS FOR INTERVENTIONAL TRIALS

SPIRIT 2013 Checklist: Recommended items to address in a clinical trial protocol and related documents*

Section/item	Item No	Description	Page Number on which item is reported
**Administrative information**			
Title	1	Descriptive title identifying the study design, population, interventions, and, if applicable, trial acronym	Page 1
Trial registration	2a	Trial identifier and registry name. If not yet registered, name of intended registry	Page 3
	2b	All items from the World Health Organization Trial Registration Data Set	Throughout the paper checked
Protocol version	3	Date and version identifier	Page 10
Funding	4	Sources and types of financial, material, and other support	Page 11
Roles and responsibilitie s	5a	Names, affiliations, and roles of protocol contributors	Page 11
	5b	Name and contact information for the trial sponsor	Page 11
	5c	Role of study sponsor and funders, if any, in study design; collection, management, analysis, and interpretation of data; writing of the report; and the decision to submit the report for publication, including whether they will have ultimate authority over any of these activities	Page 11
	5d	Composition, roles, and responsibilities of the coordinating centre, steering committee, endpoint adjudication committee, data management team, and other individuals or groups overseeing the trial, if applicable (see Item 21a for data monitoring committee)	Page 9 and 10
**Introduction**			

For peer review only

Background and rationale	6a	Description of research question and justification for undertaking the trial, including summary of relevant studies (published and unpublished) examining benefits and harms for each intervention	Page 4
	6b	Explanation for choice of comparators	Page 4-5
Objectives	7	Specific objectives or hypotheses	Page 4
Trial design	8	Description of trial design including type of trial (eg, parallel group, crossover, factorial, single group), allocation ratio, and framework (eg, superiority, equivalence, noninferiority, exploratory)	Page 5
**Methods: Participants, interventions, and outcomes**			
Study setting	9	Description of study settings (eg, community clinic, academic hospital) and list of countries where data will be collected. Reference to where list of study sites can be obtained	Page 5
Eligibility criteria	10	Inclusion and exclusion criteria for participants. If applicable, eligibility criteria for study centres and individuals who will perform the interventions (eg, surgeons, psychotherapists)	Page 5
Interventions	11a	Interventions for each group with sufficient detail to allow replication, including how and when they will be administered	Page 5-7
	11b	Criteria for discontinuing or modifying allocated interventions for a given trial participant (eg, drug dose change in response to harms, participant request, or improving/worsening disease)	Page 7
	11c	Strategies to improve adherence to intervention protocols, and any procedures for monitoring adherence (eg, drug tablet return, laboratory tests)	Page 9
	11d	Relevant concomitant care and interventions that are permitted or prohibited during the trial	Page 5
Outcomes	12	Primary, secondary, and other outcomes, including the specific measurement variable (eg, systolic blood pressure), analysis metric (eg, change from baseline, final value, time to event), method of aggregation (eg, median, proportion), and time point for each outcome. Explanation of the clinical relevance of chosen efficacy and harm outcomes is strongly recommended	Page 7

Participant timeline	13	Time schedule of enrolment, interventions (including any run-ins and washouts), assessments, and visits for participants. A schematic diagram is highly recommended (see Figure)	Page 7
Sample size	14	Estimated number of participants needed to achieve study objectives and how it was determined, including clinical and statistical assumptions supporting any sample size calculations	Page 7
Recruitment	15	Strategies for achieving adequate participant enrolment to reach target sample size	Page 7
**Methods: Assignment of interventions (for controlled trials)**			
Allocation:			
Sequence generation	16a	Method of generating the allocation sequence (eg, computer-generated random numbers), and list of any factors for stratification. To reduce predictability of a random sequence, details of any planned restriction (eg, blocking) should be provided in a separate document that is unavailable to those who enrol participants or assign interventions	Page 8
Allocation concealment mechanism	16b	Mechanism of implementing the allocation sequence (eg, central telephone; sequentially numbered, opaque, sealed envelopes), describing any steps to conceal the sequence until interventions are assigned	Page 8
Implementation	16c	Who will generate the allocation sequence, who will enrol participants, and who will assign participants to interventions	Page 8
Blinding (masking)	17a	Who will be blinded after assignment to interventions (eg, trial participants, care providers, outcome assessors, data analysts), and how	Page 7
	17b	If blinded, circumstances under which unblinding is permissible, and procedure for revealing a participant's allocated intervention during the trial	NA
**Methods: Data collection, management, and analysis**			

Data collection methods	18a	Plans for assessment and collection of outcome, baseline, and other trial data, including any related processes to promote data quality (eg, duplicate measurements, training of assessors) and a description of study instruments (eg, questionnaires, laboratory tests) along with their reliability and validity, if known. Reference to where data collection forms can be found, if not in the protocol	Page 8
	18b	Plans to promote participant retention and complete follow-up, including list of any outcome data to be collected for participants who discontinue or deviate from intervention protocols	Page 8
Data management	19	Plans for data entry, coding, security, and storage, including any related processes to promote data quality (eg, double data entry; range checks for data values). Reference to where details of data management procedures can be found, if not in the protocol	Page 8
Statistical methods	20a	Statistical methods for analysing primary and secondary outcomes. Reference to where other details of the statistical analysis plan can be found, if not in the protocol	Page 8-9
	20b	Methods for any additional analyses (eg, subgroup and adjusted analyses)	NA
	20c	Definition of analysis population relating to protocol non-adherence (eg, as randomised analysis), and any statistical methods to handle missing data (eg, multiple imputation)	Page 8
**Methods: Monitoring**			
Data monitoring	21a	Composition of data monitoring committee (DMC); summary of its role and reporting structure; statement of whether it is independent from the sponsor and competing interests; and reference to where further details about its charter can be found, if not in the protocol. Alternatively, an explanation of why a DMC is not needed	Page 9
	21b	Description of any interim analyses and stopping guidelines, including who will have access to these interim results and make the final decision to terminate the trial	Page 9

Harms	22	Plans for collecting, assessing, reporting, and managing solicited and spontaneously reported adverse events and other unintended effects of trial interventions or trial conduct	Page 9
Auditing	23	Frequency and procedures for auditing trial conduct, if any, and whether the process will be independent from investigators and the sponsor	Page 9
**Ethics and dissemination**			
Research ethics approval	24	Plans for seeking research ethics committee/institutional review board (REC/IRB) approval	Page 11
Protocol amendments	25	Plans for communicating important protocol modifications (eg, changes to eligibility criteria, outcomes, analyses) to relevant parties (eg, investigators, REC/IRBs, trial participants, trial registries, journals, regulators)	Page 10
Consent or assent	26a	Who will obtain informed consent or assent from potential trial participants or authorised surrogates, and how (see Item 32)	Page 7
	26b	Additional consent provisions for collection and use of participant data and biological specimens in ancillary studies, if applicable	NA
Confidentiality	27	How personal information about potential and enrolled participants will be collected, shared, and maintained in order to protect confidentiality before, during, and after the trial	Page 8
Declaration of interests	28	Financial and other competing interests for principal investigators for the overall trial and each study site	Page 11
Access to data	29	Statement of who will have access to the final trial dataset, and disclosure of contractual agreements that limit such access for investigators	Page 8
Ancillary and post-trial care	30	Provisions, if any, for ancillary and post-trial care, and for compensation to those who suffer harm from trial participation	NA
Dissemination policy	31a	Plans for investigators and sponsor to communicate trial results to participants, healthcare professionals, the public, and other relevant groups (eg, via publication, reporting in results databases, or other data sharing arrangements), including any publication restrictions	Page 10

For peer review only

	31b	Authorship eligibility guidelines and any intended use of professional writers	Page 10
	31c	Plans, if any, for granting public access to the full protocol, participant-level dataset, and statistical code	Page 12
**Appendices**			
Informed consent materials	32	Model consent form and other related documentation given to participants and authorised surrogates	Attached separately
Biological specimens	33	Plans for collection, laboratory evaluation, and storage of biological specimens for genetic or molecular analysis in the current trial and for future use in ancillary studies, if applicable	NA

*It is strongly recommended that this checklist be read in conjunction with the SPIRIT 2013 Explanation & Elaboration for important clarification on the items. Amendments to the protocol should be tracked and dated. The SPIRIT checklist is copyrighted by the SPIRIT Group under the Creative Commons "Attribution-NonCommercial-NoDerivs 3.0 Unported" license.

For peer review only
