# Supplementary material for: Protocol of a randomised controlled trial of real-time continuous glucose monitoring in neonatal intensive care ‘REACT’
Source: BMJ Open. 2018 Jun 4;8(6):e020816. doi: 10.1136/bmjopen-2017-020816 (PMC5988133; doi:10.1136/bmjopen-2017-020816)
Supplement: Reviewer comments [file bmjopen-2017-020816.reviewer_comments.pdf]

PEER REVIEW HISTORY

BMJ Open publishes all reviews undertaken for accepted manuscripts. Reviewers are asked to complete a checklist review form (**http://bmjopen.bmj.com/site/about/resources/checklist.pdf**) and are provided with free text boxes to elaborate on their assessment. These free text comments are reproduced below.

ARTICLE DETAILS

**TITLE (PROVISIONAL)**	Protocol of a Randomised Controlled Trial of Real Time Continuous Glucose Monitoring in Neonatal Intensive Care 'REACT'
**AUTHORS**	Beardsall, Kathryn; Thomson, Lynn; Guy, Catherine; Weissenbruch, Mirjam; Iglesias, Isabel; Muthukumar, Priya; Somisetty, Sateesh; Bond, Simon; Petrou, Stavros; Dunger, David

This article was not externally reviewed at BMJ Open. Protocols that have been independently assessed prior to submission to BMJ Open are usually fast-tracked to publication on the grounds that further substantial changes will not be possible. This independent assessment will usually be external, independent review for both a substantial grant award from a non-commercial or government funder and ethics approval.
